# Recent Advances of Composite Nanomaterials for Antibiofilm Application

**DOI:** 10.3390/nano13192725

**Published:** 2023-10-08

**Authors:** Ruilian Qi, Yuanyuan Cui, Jian Liu, Xiaoyu Wang, Huanxiang Yuan

**Affiliations:** 1 Department of Chemistry, College of Chemistry and Materials Engineering, Beijing Technology and Business University, Beijing 100048, China; qiruilian@btbu.edu.cn (R.Q.); 2230401037@st.btbu.edu.cn (Y.C.); 2 Institute of Chemistry, Chinese Academy of Sciences, Beijing 100090, China; liujian13@iccas.ac.cn; 3 School of Materials Science and Engineering, University of Science and Technology Beijing, Beijing 100083, China; wangxy@ustb.edu.cn

**Keywords:** bacterial biofilm, organic composite nanomaterials, inorganic nanomaterials, organic–inorganic hybrid nanomaterials, permeability, antibiofilm mechanism

## Abstract

A biofilm is a microbial community formed by bacteria that adsorb on the surface of tissues or materials and is wrapped in extracellular polymeric substances (EPS) such as polysaccharides, proteins and nucleic acids. As a protective barrier, the EPS can not only prevent the penetration of antibiotics and other antibacterial agents into the biofilm, but also protect the bacteria in the biofilm from the attacks of the human immune system, making it difficult to eradicate biofilm-related infections and posing a serious threat to public health. Therefore, there is an urgent need to develop new and efficient antibiofilm drugs. Although natural enzymes (lysozyme, peroxidase, etc.) and antimicrobial peptides have excellent bactericidal activity, their low stability in the physiological environment and poor permeability in biofilms limit their application in antibiofilms. With the development of materials science, more and more nanomaterials are being designed to be utilized for antimicrobial and antibiofilm applications. Nanomaterials have great application prospects in antibiofilm because of their good biocompati-bility, unique physical and chemical properties, adjustable nanostructure, high permeability and non-proneness to induce bacterial resistance. In this review, with the application of composite nanomaterials in antibiofilms as the theme, we summarize the research progress of three types of composite nanomaterials, including organic composite materials, inorganic materials and organic–inorganic hybrid materials, used as antibiofilms with non-phototherapy and phototherapy modes of action. At the same time, the challenges and development directions of these composite nanomaterials in antibiofilm therapy are also discussed. It is expected we will provide new ideas for the design of safe and efficient antibiofilm materials.

## 1. Introduction

Infections resulting from pathogenic bacteria have become one of the main causes of human morbidity and death. It is reported that up to 1 million people fall ill or die from biofilm infections each year, and more than 60% of human pathogenic infections and 80% of chronic diseases are attributed to biofilm [1,2], such as chronic tonsillitis [3], otitis media [4], cystic fibrosis and endocarditis [5,6]. About 40–80% of bacteria in nature can form a biofilm [7], which is a microbial community formed by bacteria or fungi adsorbed on the surface of tissues or materials and wrapped in extracellular polymeric substances (EPS) such as polysaccharides, proteins and nucleic acids. EPS and encapsulated microbial communities comprise 82–85% and 15–20%, respectively [8]. As a protective barrier, EPS can not only prevent the penetration of antibiotics and other antibacterial agents into the membrane, but also protect the bacteria in the biofilm from the attack of the human immune system [1], thus making the related diseases caused by the biofilm difficult to cure. In addition, a key reason for bacterial resistance is the formation of bacterial biofilms [9]. Compared with ordinary bacterial biofilms, the biofilms of multidrug-resistant bacteria are more difficult to eradicate, posing a serious threat to public health. In order to cure serious biofilm infections, it is necessary to explore and develop highly effective antibacterial drugs that can effectively inhibit and eradicate biofilms.

Antibacterial agents such as natural enzymes (lysozyme, peroxidase, etc.) and antimicrobial peptides have broad-spectrum antibacterial activity [10], which can effectively inhibit the growth and reproduction of a variety of pathogenic microbes (bacteria, fungi, etc.) [11], such as *Escherichia coli*, *Staphylococcus aureus*, *Pseudomonas aeruginosa* and *Candida albicans*. Different from synthetic antimicrobials that face the problems of side effects and drug resistance, natural antimicrobial agents have the characteristics of low toxicity and no side effects [12]. Therefore, antibacterial agents such as natural enzymes and antimicrobial peptides have become potential substitutes for traditional antibiotic therapy. Miriam Kalsy et al. [13] demonstrated that the insect antimicrobial peptide CecA can inhibit the formation of *E. coli* biofilm through the multi-target mechanism of inducing a change in bacterial outer membrane permeability, thus inhibiting efflux pump activity and nucleic acid interaction inside and outside the bacterial membrane. This mechanism ensures that it can greatly delay the development of bacterial resistance in combination with antibiotic niridinic acid. Jasmin Portelinha et al. [14] demonstrated that a metal antimicrobial peptide, Gaduscidin-1, with a multi-action mode (nuclease activity for cutting eDNA in biofilms) can eradicate *P. aeruginosa* biofilm. In addition, when combined with kanamycin, it has a synergistic antibacterial effect and is a potential anti-biological film agent for the treatment of cystic fibrosis caused by *P. aeruginosa* biofilm. In addition, antimicrobial peptides such as Cec4 [15], piscidin [16] and CRAMP [17] are also successfully used in the field of antibiofilms. Although these natural antimicrobial agents have excellent bactericidal activities, they exhibit disadvantages including low stability in physiological environments, short antibacterial durations and poor permeability in biofilms [18], which limit their application in combatting biofilms.

Nanocomposites have great application prospects in antibiofilms due to their advantages of good biocompatibility, unique physical and chemical properties and adjustable nanostructures [19], and they do not easily develop bacterial drug resistance. Antimicrobials, metal compounds and photosensitizers have been modified by biocompatible chitosan, polyvinyl alcohol (PVA) and other materials to improve the solubility, stability, permeability and dispersion properties of the materials, and this then improved the antibacterial or antibiofilm activity [20,21,22,23,24]. The POD-like catalytic activity of cobalt single-atomic nanozymes could be improved by adjusting the number of nitrogen coordination sites (Nx = 2, 3, 4) [25]. At present, different kinds of nanomaterials have been gradually applied to combat biofilms, including organic and inorganic nanomaterials loaded with antibiotics [26,27], polymer-modified nanomaterials [28,29], metal and metal oxide nanomaterials [30,31,32], and nano-enzymes [33,34]. Most nanocomposites can achieve synergistic antibacterial effects in the following ways: (1) the positive charge on the surface of the materials can be electrostatically bonded to the negatively charged bacterial surface to improve the targeting of drugs; (2) nanomaterials serve as carriers to deliver antimicrobials to the biofilm and increase the accumulation of drugs in the biofilm; (3) nanocomposites achieve the eradication of biofilms through phototherapy (photodynamic and photothermal therapies) and non-phototherapy (chemotherapy and physical damage, etc.) treatment modes [35]. Among these, photodynamic therapy (PDT) is a process by which photosensitizers transfer energy to surrounding oxygen after being excited by light, and then produce toxic reactive oxygen species (ROS), which cause irreversible damage to bacteria and further induce death. Photothermal therapy (PTT) is a process in which a photothermal agent converts light energy into heat and thermally ablates bacteria. PDT and PTT are widely used sterilization strategies in the fields of antibacterial and antibiofilm applications due to their advantages of flexible controllability, low toxicity, less invasiveness, high specificity, and low capacity for developing resistance. The non-phototherapy mode can exert antibacterial effects without external energy input, and has great application prospects in antibiofilms. However, the efficiency of a single mode for antibiofilms is often low, and the combination of multiple materials can achieve the efficient multi-mode removal of a biofilm. Importantly, based on nanomaterials, intelligent antibacterial platforms can be designed in response to biofilm microenvironment (pH, GSH, H_2_O_2_, etc.) and external stimuli (light, electricity, magnetic, heat, sound, etc.) that can release drugs under specific stimuli or induce collaborative antibacterial mechanisms, which has attracted great attention in biofilm research [36].

This review summarizes in detail the research progress of nanocomposites in the field of antibiofilms made in the past three years, and introduces the antibiofilm effect and possible mechanism of organic nanocomposites, inorganic nanomaterials and organic–inorganic hybrid nanomaterials (binary and ternary hybrid materials), which are synthesized by non-covalent interactions or covalent connections. The challenges and development directions of three kinds of nanomaterials used in treating biofilm are also discussed. It is expected that the review could help researchers develop more effective antibiofilm agents to address serious biofilm infections.

## 2. Organic Nanocomposites

Organic nanocomposite antibacterial materials usually refer to composite nanoparticles with different physical properties [37] (charge and size, etc.), which are assembled by the covalent connection or non-covalent interaction of one or more antibacterial organic functional molecular components. According to their functions, the component molecules can be divided into two main categories: (a) component units with antibacterial properties themselves, which can be a single antibacterial unit or a combination of multiple antibacterial units [38,39,40]; (b) components that improve the physical properties of materials (water solubility, charge and size, etc.), introduce stimulus-response mechanisms, and act as drug carriers [41,42]. The organic antibacterial nanocomposite constructed thus may reduce biofilm adhesion, increase the permeability of antibiofilm agents, improve drug stability and targeting [2], and combined with treatment modes such as CDT, PDT and PTT, show significant antibacterial and antibiofilm effects, and can thus accelerate wound healing. In this section, the organic nanocomposites constructed by non-covalent interaction and covalent connection are classified, and the research progress made into organic nanocomposites in the field of antibiofilms in the past three years is introduced.

### 2.1. Construction of Organic Nanocomposites by Non-Covalent Interaction

The non-covalent interaction between various functional molecules, such as host–guest interaction, electrostatic interaction, the π–π conjugated effect and hydrophobic interaction, can be used to construct organic nanocomposites with stimulation-response and multifunctional integration capacities. Because these weak interactions are sensitive to external conditions (pH, temperature, ionic strength, etc.), the material can be imparted with stimulus responsiveness. A multifunctional integrated nanoplatform can be constructed by combining multiple functional molecules. This section classifies organic nanocomposites based on host–guest interactions and other non-covalent interactions, and elaborates the research into and applications of these two types of materials for antibiofilm use.

#### 2.1.1. Host–Guest Interaction to Construct Organic Nanocomposites

Host–guest interaction mainly refers to the process by which macrocyclic molecules (cyclodextrin, cucurbit[n]urils and calysarene, etc.) and guest molecules combine to form supramolecules through non-covalent interaction on the basis of complementary shape and size [43]. Host–guest interactions usually have a high binding constant [44], and supramolecular assemblies with different morphologies and functions can be constructed by selecting different guest molecules. Therefore, supramolecules constructed based on the host–guest interaction have attracted great attention in the field of biomedicine.

**Figure 1:**
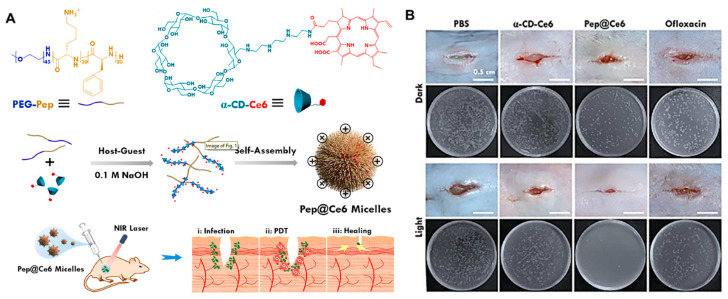
(**A**) Pep@Ce6 micelle construction and schematic diagram of photodynamic treatment of wound infection caused by *P. aeruginosa* biofilm. (**B**) Effects of Pep@Ce6 micelles on bacterial wound infection after different treatments. Reprinted with permission from Ref. [45]. Copyright 2021 Elsevier Ltd.

By connecting the photosensitizer and the positively charged group on the host and the guest, respectively, a bifunctional supramolecular assembly with the ability to target the negatively charged bacteria and enact photodynamic killing can be formed after self-assembly. Guosheng Fu et al. [45] formed a host–guest complexation between α-cyclodextrin (α-CD-Ce6) modified with the photosensitizing agent dihydroporphyrin (Ce6) and polyethylene glycol acylated polypeptide (PEG-Pep) to construct a photodynamic therapy platform (Pep@Ce6) for the eradication of *P. aeruginosa* biofilm and the treatment of wounds (Figure 1A). Positively charged Pep@Ce6 micelles can bind to lipopolysaccharides of the outer membrane of *P. aeruginosa* and destroy the integrity of the membrane. Under NIR irradiation, Pep@Ce6 micelles produce more singlet oxygen than α-CD-Ce6, which has PDT bactericidal potential. In vitro antibacterial experiments have shown that the MIC of Pep@Ce6 micelles against *P. aeruginosa* under light irradiation was 64 μg·mL^−1^, which was significantly lower than that in a dark environment (>256 μg·mL^−1^) and that of the α-CD-Ce6 group (light or dark, 256 μg·mL^−1^). Compared with α-CD-Ce6, which could not damage the bacterial film in time due to the short ROS generation lifetime and action range, Pep@Ce6 micelles were more effective in recognizing bacteria and producing ROS in situ, thus showing a significant bactericidal rate. In in vitro antibacterial experiments, the Pep@Ce6 micellar treatment reduced the thickness of biofilm from 25 μM to 14 μM, and damaged the integrity of the bacterial membrane, indicating that it has a good biofilm penetration and clearance ability. The viability of 3T3-L1 cells (a preadipocyte cell line) treated with 200 μg·mL^−1^ of Pep@Ce6 was above 80% regardless of light exposure, showing good cell compatibility. At the same time, the MIC value of Pep@Ce6 micelles against *P. aeruginosa* within 10 generations was almost unchanged, which has the potential to alleviate the generation of drug resistance in bacteria. In a mouse model of a knife wound, the NIR/Pep@Ce6 micellar treatment group almost completely removed bacteria from the wound, effectively inhibited inflammatory cell infiltration, and accelerated wound healing (Figure 1B). The cationic Pep@Ce6 micelle is an effective antibiofilm agent for the treatment of *P. aeruginosa* biofilm infection.

**Figure 2:**
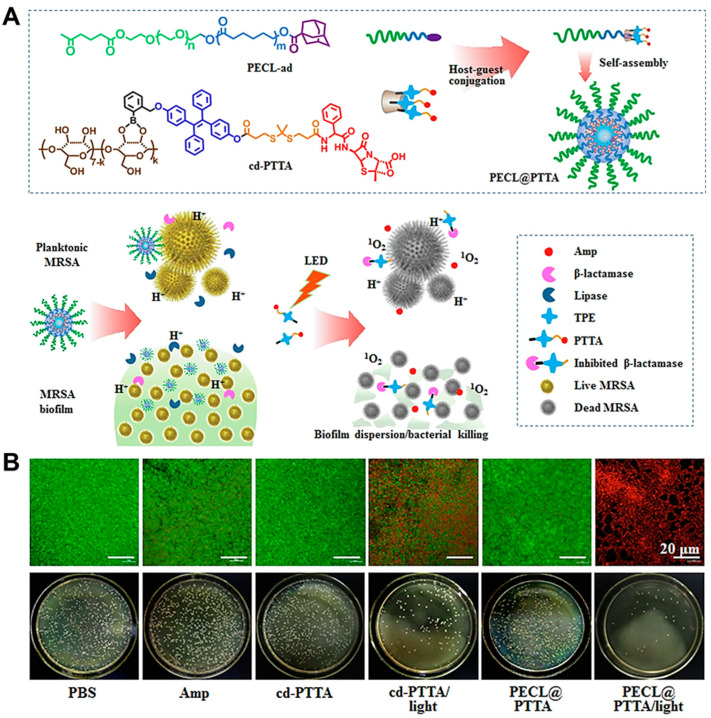
(**A**) Schematic diagram of the antibacterial mechanism of PECL@PTTA. (**B**) Staining fluorescence microscope images of live and dead bacteria in an MRSA biofilm and plates of MRSA colonies remaining in the biofilm after different treatments. Reprinted with permission from Ref. [46]. Copyright 2021 Elsevier Ltd.

Although drugs can be targeted to identify bacteria through electrostatic interactions, a higher ionic strength will greatly reduce the electrostatic binding effects of drugs and bacteria in physiological environments. In addition, the biofilm microenvironment is characterized by low oxygen content, weak acidity, and a high concentration of GSH and H_2_O_2_, which often make antimicrobials ineffective. To improve the efficient and targeted killing of bacteria using drug molecules in biofilms, Maohu Chen and colleagues [46] designed a light-triggered and acid-responsive chemotherapy/PDT synergistic antibacterial platform (PECL@PTTA) for antimicrobial and antibiofilm use. The collaborative antibacterial platform was formed from the host–guest complex of the hydrophilic fragment PECL-ad and the hydrophobic fragment cd-PTTA (Figure 2A). The hydrophilic fragment PECL-ad is synthesized by the terminal connection of poly (ethylene glycol)-poly (ε-caprolactone) to adamantane, and the hydrophobic fragment cd-PTTA is covalently linked by five functional units including the main molecule β-cyclodextrin, β-lactamase inhibitor phenylboric acid (PBA), photosensitizer TPE, ROS-responsive thione linker and antibiotic ampicillin (Amp). Under weakly acidic (pH 5.5) conditions, the sizes of micelles gradually increase with time due to borate cracking, which leads to the removal of β-CD from cd-PTTA and the enhancement of hydrophobicity, followed by an increase in the aggregation size. The exposed phenylboric acid deactivates β-lactamase, and then the sensitivity of bacteria to Amp restores. At the same time, PECL@PTTA micelles are enzyme-responsive. After the supramolecular micelles spread to the biofilm, polycaprolactone fragments are degraded by bacterial lipase, and the prodrug cd-PTTA is released. PECL@PTTA micelles are also photo-responsive. Under the irradiation of NIR light, TPE in cd-PTTA can be excited to produce ROS, and then break the thione link to release Amp. These results suggest that PECL@PTTA micelles respond to multiple stimuli (pH, lipase, NIR). In in vitro antibacterial experiments, compared with Amp (32 μg·mL^−1^) with the lowest bactericidal activity against MRSA, 16 μg·mL^−1^ of PECL@PTTA micelle was shown to significantly inhibit the growth and reproduction of bacteria under pH 5.5 and NIR irradiation, showing excellent antibacterial activity with a bactericidal rate of more than 82%. In in vitro antibiofilm experiments, the PECL@PTTA/NIR group effectively dispersed the biofilm of MRSA with a biofilm clearance rate of 83% (Figure 2B). In a model of subcutaneous abscess caused by MRSA biofilm infection, the PECL@PTTA/NIR group killed 87% of the bacteria at the wound site, and promoted vascular regeneration and wound epithelialization for accelerating wound healing. The system has good biocompatibility and is almost non-toxic to cells, even at concentrations as high as 800 μg·mL^−1^. Overall, the photo-triggered and pH/lipase-responsive PECL@PTTA platform provides a new strategy for restoring antibiotic susceptibility to resistant bacteria and efficiently clearing biofilms.

**Figure 3:**
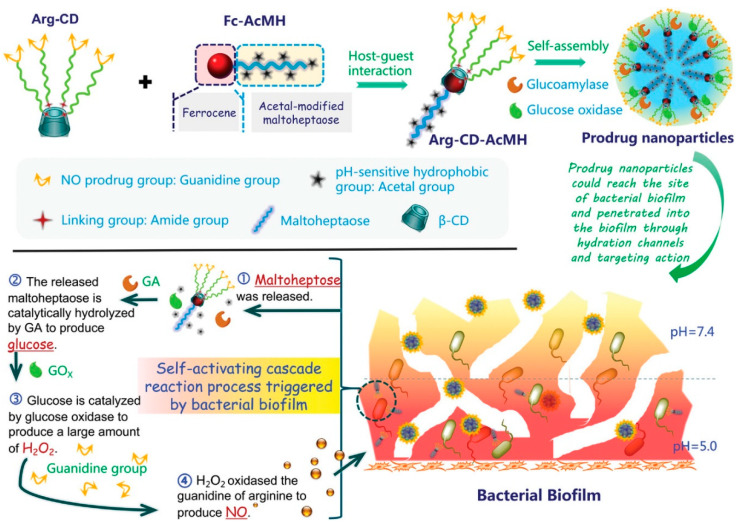
Schematic diagram of the cascade catalysis mechanism of the self-activated antibacterial nanoplatform Arg-CD-AcMH. Reprinted with permission from Ref. [47]. Copyright 2022 Wiley-VCH GmbH.

In addition, because the gas molecules NO and CO not only have broad-spectrum antibacterial activity, but also have good permeability into biofilms, they have been gradually applied in antibiofilm research in recent years. Guanghui Zhao’s group [47] designed a self-activated antimicrobial nanoparticle (Arg-CD-AcMH) for antibiofilm therapy via the host–guest interaction between L-arginine (hydrophilic fragment) modified with β-CD and acetalized maltoheptaose modified with ferrocene (hydrophobic fragment) by wrapping glucose oxidase and glucose amylase via the process of self-assembly (Figure 3). The nanoparticles are acid-responsive and can release NO following self-activation in a slightly acidic environment. In the biofilm environment, which is mildly acidic, the acetal bond on maltoheptaose is broken, resulting in the dissociation of the nanoparticles, and the released maltoheptaose is hydrolyzed by glucose amylase to produce glucose that is catalyzed by glucose oxidase to produce H_2_O_2_, followed by oxidizing the guanidine group of arginine to produce antibacterial NO molecules, thus causing irreversible damage to bacteria. In in vitro antibacterial experiments, under acidic conditions, 0.5 mg·mL^−1^ of Arg-CD-AcMH/GOx/GA can kill more than 95% of *E. coli* and *S. aureus*. In addition, the nanoparticle has a good biofilm penetration ability. After entering the biofilm, it can be destroyed by the continuous release of NO through the enzymatic cascade catalytic effect, and biofilms formed by *E. coli* and *S. aureus* can be almost completely eliminated. In the treatment of wound infection, Arg-CDAcMH/GOx/GA can also reduce inflammatory response, promote collagen deposition and new angiogenesis, and accelerate rapid wound healing. This study provides a new outlook for developing a novel self-activation response platform.

**Figure 4:**
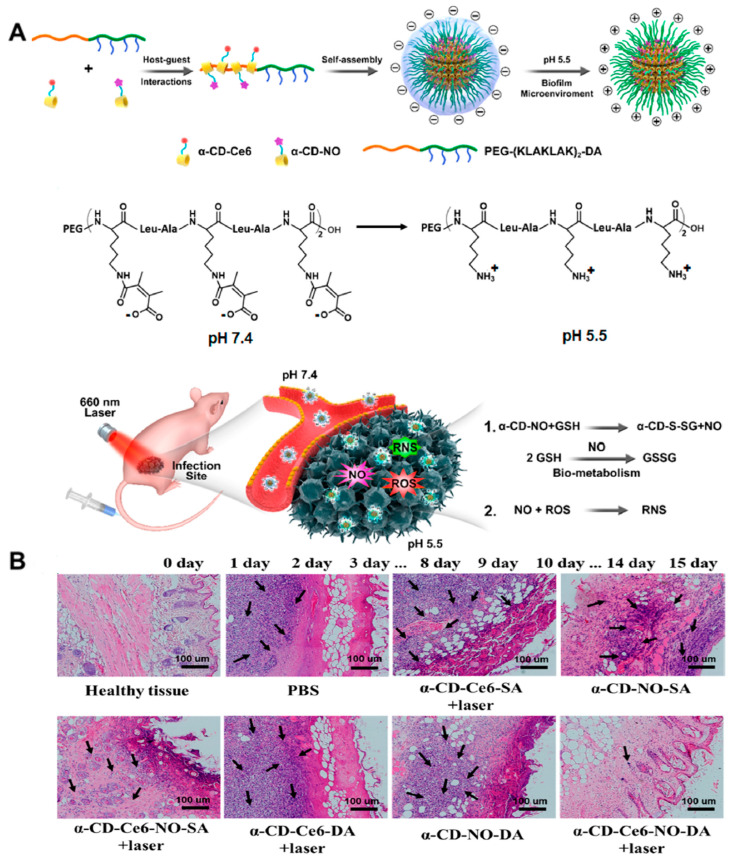
(**A**) Schematic diagram of the antibacterial mechanism of the supramolecular nanocarrier α-CD-Ce6-NO-DA. (**B**) Tissue microscope images (0.2 W·cm^−2^, 1 min) of infected sites treated with different nanocarriers, indicating inflammatory cells at the black tip (30 days after treatment). Reprinted with permission from Ref. [1]. Copyright 2020 American Chemical Society.

In order to further improve the antibiofilm effect of NO, Yifan Gao et al. [1] reported the use of an acid-responsive supramolecular nanocarrier (α-CD-Ce6-NO-DA) with charge-switched properties for biofilm removal through the NO/PDT synergistic mode. α-CD-CE6-no-DA consists of a GSH-sensitive NO-prodrug (α-CD-NO), a photosensitizer Ce6-prodrug (α-CD-Ce6), and a pH-sensitive polypeptide copolymer (PEG-(KLAKLAK)_2_-DA) (Figure 4A). After reaching the biofilm, the acidic environment caused the surface charge of the nanocarrier to change from negative to positive, enhancing the targeted identification of the biofilm and the penetration into the biofilm. After penetrating into the biofilm, the high level of GSH will cause α-CD-NO to release NO rapidly, which can kill bacteria and reduce the level of GSH in the biofilm. Under visible light (660 nm) irradiation, Ce6 catalyzes the generation of ROS, and ROS reacts with NO to generate reactive nitrogen species (RNS) with higher oxidation activity, further improving the PDT effect. In vitro antibacterial experiments have shown that under visible light irradiation (0.2 W·cm^−2^, 1 min), the amount of α-CD-Ce6-NO-DA required to achieve a 99.9% bactericidal rate contained 10 μg·mL^−1^ equivalent Ce6 and 20 μg·mL^−1^ equivalent NO, which is 1/16 of the dose of α-CD-Ce6-DA, without the loading of a NO precursor. Under the same conditions, α-CD-Ce6-NO-DA shows lower cytotoxicity. In a mouse model of MRSA infection, the nanocarrier effectively concentrated in the region of biofilm infection, effectively removed bacteria and biofilm from the wound site, and reduced the number of inflammatory cells for rapid wound healing (Figure 4B). In conclusion, the pH-responsive NO/PDT collaborative system can quickly remove biofilms at low photosensitizer doses and laser intensity, providing guidance for the design of gas collaborative antibacterial strategies.

#### 2.1.2. Construction of Organic Nanocomposites Based on Other Non-Covalent Interactions

Antibacterial agents can bind to other functional molecules or carrier molecules through non-covalent interactions such as electrostatic interaction, hydrogen bonding, hydrophobic interaction and π–π interaction, and self-assemble to form nanoparticles to achieve the purpose of delivering natural antibacterial agents, antibiotics, photosensitizers, and proteases.

**Figure 5:**
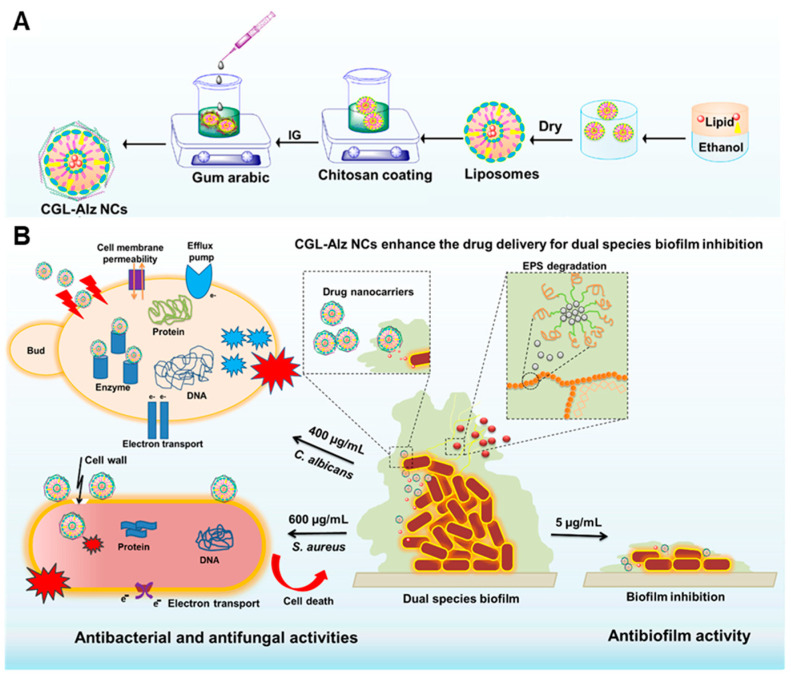
(**A**) Schematic diagram of the preparation of CGL-Alz NCs. (**B**) Illustration of possible mechanisms of antimicrobial/antifungal activities. Reprinted with permission from Ref. [48]. Copyright 2021 Elsevier Ltd.

Water solubility can be improved effectively by wrapping the natural antibacterial agent within the liposome, and the continuous release of the natural antibacterial agent can be achieved by modifying the biopolymer through electrostatic interactions on the surface. Vinit Raj et al. [48] synthesized chitosan–gum arabic-coated liposome alizarin nanocarriers (CGL-Alz NCs) using the ionic gel method to inhibit the formation of a variety of microbial biofilms. CGL-Alz NCs has high stability and can protect alizarin from oxidation. The positive surface charge can improve the penetration efficiency of alizarin in biofilm and its targeting to the bacterial membrane. Compared with alizarin, GL-ALz NCs significantly inhibited the growth of *C. albicans* and *S. aureus* (Figure 5A). In in vitro antibiofilm experiments, the rate of inhibition of *C. albicans* and *E. coli* biofilm formation by CGL-Alz NCs reached 90%. In addition, 50 μg·mL^−1^ of CGL-Alz NCs inhibited the biofilm formation of two microbes by 95%, and the CGL-Alz NCs effectively reduced the thickness of single or dual biofilms. Furthermore, 20 μg·mL^−1^ of CGL-Alz NCs was shown to significantly inhibit the formation of *C. albicans* mycelia and thus inhibit the formation of its biofilm. The possible antimicrobial mechanism involves the entry of nanocarriers into microbes, which may lead to protein inactivation, interaction with nucleic acids (DNA and RNA), the alteration of efflux pumps, and the destruction of membrane integrity (Figure 5B). This paper reports for the first time that the designed CGL-Alz NCs have antibiofilm activity. The combination of the NC and alizarin provides a new strategy for combating fungi and bacteria, and their single and dual biofilms.

PDT has excellent antibacterial capacity, but the killing efficiency of PDT is usually low due to the hypoxic state of the biofilm microenvironment. Therefore, the antibiofilm effect of PDT can be significantly enhanced by simultaneously delivering a photosensitizer and oxygen to the biofilm infection site. Lingyun Zou et al. [49] reported a liposome encapsulated with an O_2_ carrier (perfluorohexane (PFH)) and a photosensitizer (Ce6 (Lip-Ce6-PFH@O_2_)) for enhancing the bacterial biofilm clearance effect of PDT. Lip-Ce6-PFH@O_2_ has a high encapsulation rate of 94.16% for Ce6, which achieves good penetration in biofilm, and can evenly penetrate into the *P. aeruginosa* biofilm, which can significantly increase the effective concentration of the photosensitization agent in the biofilm. At the same time, after the encapsulation of an oxygen carrier, the output of singlet oxygen under light is obviously increased, and reaches 1.5 times that of Lip-Ce6. In in vitro antibacterial experiments, under NIR irradiation, the biofilm eradication rate of Lip-Ce6-PFH@O_2_ treatment is 90.1%. The biofilm eradication rate of Lip-Ce6-PFH@O_2_ is twice that of Lip-Ce6 without the encapsulation of an oxygen carrier. Lip-Ce6-PFH@O_2_ transports oxygen to the biofilm, alleviating its oxygen-deficient state. The increase in oxygen content is conducive to the generation of large amounts of ROS, thereby significantly enhancing the antibiofilm performance of PDT. In the mouse model of subcutaneous abscesses, Lip-Ce6-PFH@O_2_ can effectively remove 90% of the bacteria in the infected area under NIR light irradiation. After treatment with Lip-Ce6-PFH@O_2_, the wound tissue is benign and almost no scar is left. This indicates that it has a good biofilm eradication effect in vivo. In addition, Lip-Ce6-PFH@O_2_ has low toxicity and good biosafety. These results suggest that oxygenation strategies targeting the biofilm microenvironment can provide new ideas for the design and development of effective PDT platforms to eradicate biofilms.

**Figure 6:**
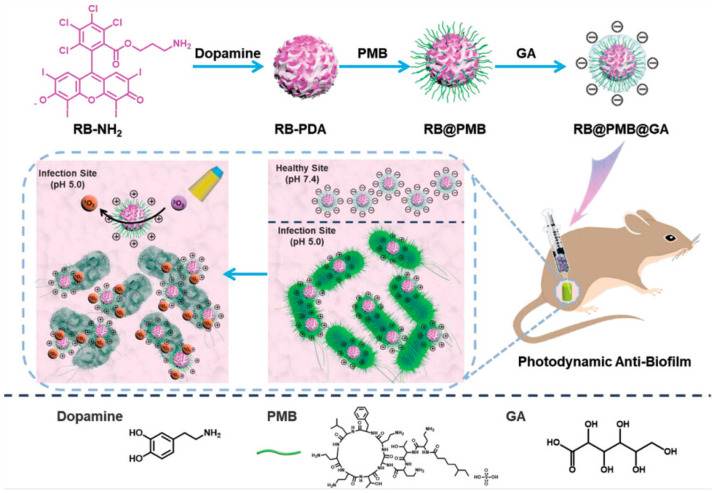
Schematic diagram of the antibacterial mechanism of RB@PMB@GA NPs. Reprinted with permission from Ref. [50]. Copyright 2021 Wiley-VCH GmbH.

Because ROS produced by PDT has a short half-life and limited diffusion radius, the antibacterial effect of PDT is limited. Therefore, improving the binding strength of nanoparticles and bacteria is more conducive to improving the antibacterial and antibiofilm properties of PDT. Shuangmei Wu et al. [50] designed a pH-sensitive photodynamic collaborative system (RB@PMB@GA NPs) for cleaning bacterial biofilms. Amylated Rose Bengal (RB-NH_2_) and polydopamine (PDA) covalently bind to generate a nanoparticle core (RB-PDA), which is functionalized by polymyxin B (PMB) and gluconic acid to form an acid-sensitive photodynamic therapy platform (RB@PMB @GANPs) (Figure 6). After arriving at the biofilm, the acid response changes the surface charge of RB@PMB@GA NPs from negative to positive, promoting the targeted recognition of the nanoparticles toward bacteria. Under NIR irradiation, the photosensitization agent RB produces ROS, and PMB binds to the outer membrane of Gram-negative bacteria, resulting in instability in the membrane, thus improving the sensitivity of bacteria to photodynamics and increasing the antibacterial effect of PDT. At the same time, an increase in local temperature can kill part of the bacteria, so as to achieve the purpose of collaborative antibacterial PDT/PTT (Figure 6). In in vitro antibacterial experiments, RB@PMB@GA NPs/NIR effectively killed *S. aureus* in both normal and acidic environments. Only under acidic conditions did RB@PMB@GA NPs have a good bactericidal effect on *E. coli*. Under acidic conditions, the MIC value of RB@PMB@GA NPs for *P. aeruginosa* was 4 mM. These results indicate that Gram-positive bacteria were more sensitive to PDT than Gram-negative bacteria, and RB@PMB@GA NPs showed broad-spectrum antibacterial properties. In in vitro antibiofilm experiments, under NIR irradiation, RB@PMB@GA NPs almost eradicated a *P. aeruginosa* biofilm at pH 5.0. In a mouse implant catheter model, the RB@PMB@GA NPs/NIR treatment group almost killed both bacteria on the implanted catheter and bacteria at the wound infection site. Moreover, 256 µM of RB@PMB@GA NPs did not cause significant hemolysis and cytotoxicity, regardless of light exposure, indicating better biocompatibility. This work provides a promising approach to treating infections associated with medical devices.

**Figure 7:**
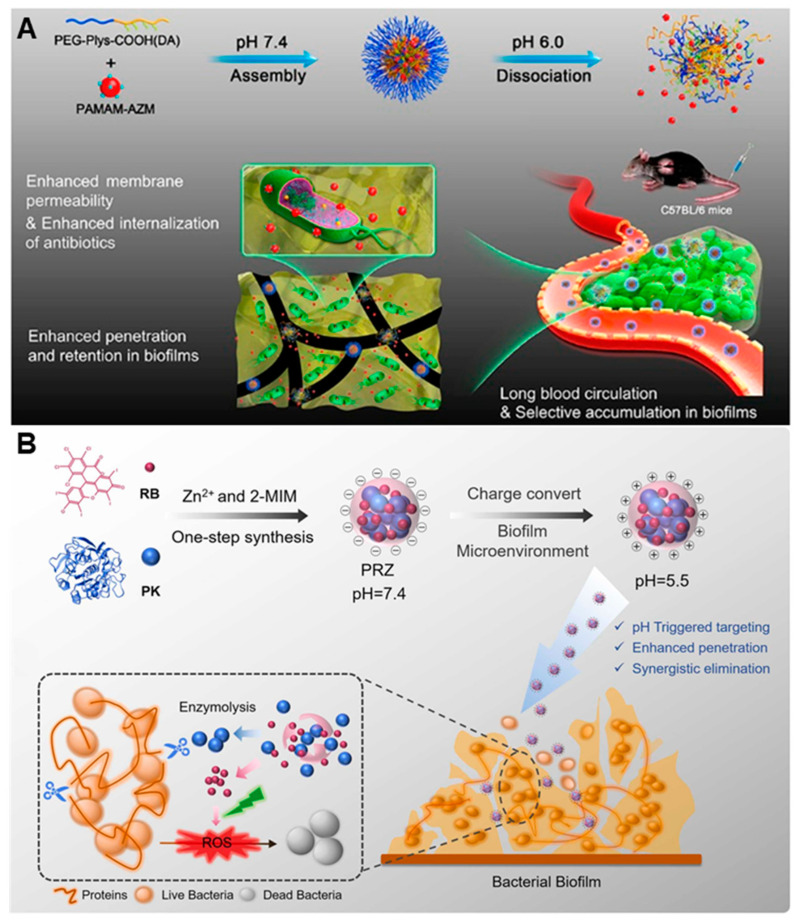
(**A**) Schematic diagram of AZM-DA NPs with acid-responsive characteristics for antibiofilm in vivo. Reprinted with permission from Ref. [51]. Copyright 2020 American Chemical Society. (**B**) Illustration of the preparation of PRZ for use in a synergistic antibiofilm of enzyme and PDT. Reprinted with permission from Ref. [52]. Copyright 2022 Elsevier Ltd.

Antibiotics are approved for clinical application, and improving their permeability on biofilms makes them more promising for clinical application. Jian Ji’s group [51] designed size- and charge-adaptive azithromycin (AZM)-modified polymer nanoparticles (AZM-DA NPs) for the treatment of chronic pulmonary infection caused by *P. aeruginosa*. The nanoparticles (NPs) were prepared by the electrostatic complexation of AZM-conjugated poly(amidoamine) dendrimer (PAMAM-AZM) with PEG-b-PLys (polyethylene glycol polylysine) modified by 2, 3-dimethylmaleic anhydride (DA). AZM-DA NPs are acid-responsive, and when they reach the slightly acidic environment of the biofilm, the charge changes from negative to positive, and smaller secondary PAMAM-AZM NPs are released, thus achieving effective penetration and long-term accumulation in the biofilm. Positively charged PAMAM-AZM NPs adhere to bacterial membranes, increase membrane permeability and promote the internalization of AZM, resulting in the efficient killing of bacteria and the efficient removal of biofilms (Figure 7A). The bactericidal rate of AZM-DA NPs was 99.994% under acidic condition. Moreover, AZM-DA NPs can significantly inhibit the levels of three virulent factors, including elastase, lipase and protease, and then inhibit the invasion of bacteria into normal cells and tissues, thus easing the evolution of infectious diseases. In in vitro antibiofilm experiments, the bactericidal rate of AZM-DA NPs against bacteria in biofilm was 99.998% with AZM equivalent of 400 μg·mL^−1^. In the mouse model of chronic lung infection, AZM-DA NPs efficiently accumulated and were retained at the site of infection, and AZM-DA NPs with an AZM equivalent of 25 μg·mL^−1^ had a bactericidal rate of 99.7%, which is twice that of the free AZM group. During treatment, the pulmonary alveolar structure of mice did not show fibrosis, and there was no inflammatory cell infiltration. There was no significant effect on the body weight of mice. Even at a high concentration of 400 μg·mL^−1^, AZM-DA NPs are non-toxic to cells and show excellent biocompatibility. More importantly, after the AZM-DA NPs treatment, the genes related to bacterial drug resistance (including *mexA*, *mexB*, *oprM*, *mexD*, *muxA*, *mexC*, *mexE* and *muxB*) were not upregulated, effectively alleviating the development of antibiotic resistance. The nanoparticles designed in this study, with the ability to target the biofilm microenvironment, provide a new idea for reducing bacterial resistance and improving the treatment of biofilm infections.

It is difficult for antibacterial agents to exert therapeutic effects in biofilms because of the obstruction effect of the extracellular matrix on drug penetration. Designs that enable the targeted decomposition of EPS is one of the most important strategies related to antibiofilms. Meng Ding et al. [52] encapsulated protease K and photosensitizer RB into a zeolite imidazolate framework (ZIF-8) to prepare a pH-responsive nanocomplex (PRZ) for bacterial biofilm eradication. Acidic environments or acidic conditions change the surface charge of the nanocomplex from negative to positive when it reaches the biofilm, promoting its penetration into the biofilm, then inducing the decomposition of the nanocomplex, releasing protease K and photosensitizer RB. Under visible light irradiation, a large amount of ROS is produced, and protease K degrades the EPS component of the biofilm, destroying its dense structure (Figure 7B). In in vitro biofilm eradication experiments, the biofilm eradication rate of PRZ in the dark only increased to 80% in the concentration range of 0–100 μg·mL^−1^. Under visible light irradiation, the biofilm eradication rate reached 95% in the concentration range of 60–100 μg·mL^−1^, indicating the synergistic bactericidal effect of PDT. In a mouse infection model, PRZ reduced the bacterial population by 5.18 × 10^8^ CFU under visible light irradiation. The wound surface and abscess were significantly reduced without inflammatory cell infiltration, and a large number of collagen fibers were formed. In addition, PRZ did not cause pathological damage to the muscle, heart, liver, spleen, lung, or kidney, exhibiting good biocompatibility. This pH-responsive enzyme/PDT synergistic strategy provides a non-antibiotic strategy for eliminating biofilms.

**Figure 8:**
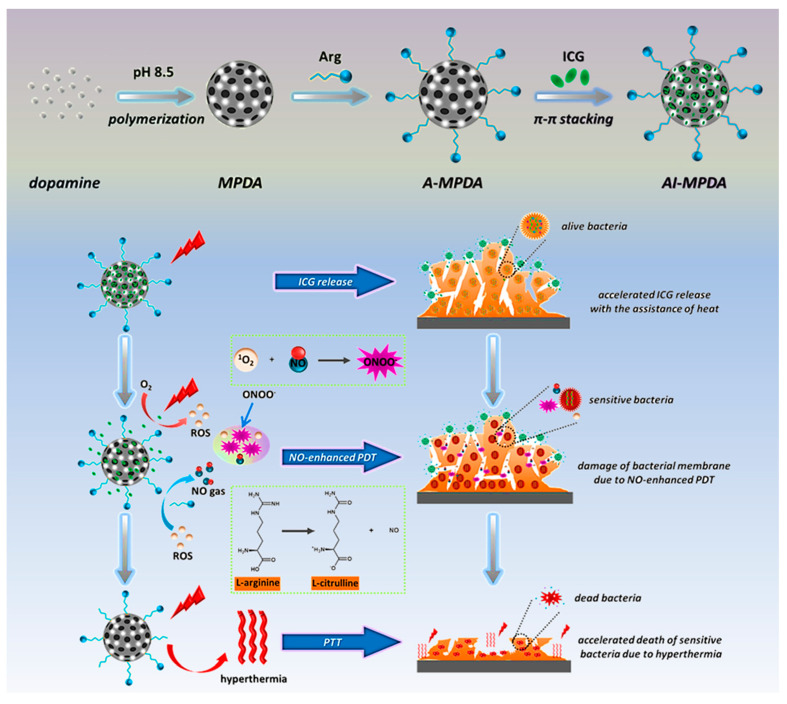
Preparation of AI-MPDA for biofilm elimination under near-infrared light with NO-enhanced low-temperature PTT. Reprinted with permission from Ref. [53]. Copyright 2020 American Chemical Society.

In order to further improve the efficiency of the antibiofilm, a multi-mode collaborative antibacterial strategy is one of the most important research directions for developing antibiofilm agents. Kaiyong Cai and colleagues [53] constructed a PTT and NO-enhanced PDT synergistic antibacterial phototherapy platform (AI-MPDA) for biofilm eradication. L-Arg reacted with MPDA via Michael addition to synthesize A-MPDA, and then AI-MPDA was constructed from A-MPDA and ICG through π–π stacking. Under NIR irradiation, the MPDA in AI-MPDA induces a local temperature increase, which promotes the release of ICG and the generation of ROS, and ROS oxidizes L-Arg to produce NO. In this process, the combined action of high temperature, ROS and NO destroys the integrity of the bacterial membrane and leads to bacterial death (Figure 8). In in vitro antibacterial experiments, the eradication rate of the AI-MPDA+NIR group applied to the *S. aureus* biofilm reached 99.0%, which was much higher than that of the I-MPDA+NIR group (58.5%), indicating that NO significantly enhanced the synergistic therapeutic effect of PDT/PTT. In the mouse skin infection model, the AI-MPDA/NIR (1.0 W·cm^−2^, 0.2 mg·mL^−1^) treatment group had a bacteriostatic rate of 90%, and the amount of inflammatory infiltration was significantly reduced, which remarkably hindered bacterial colonization at the infected site. In a wound model, AI-MPDA can eliminate almost 100% of biofilm and promote wound healing with good biocompatibility. The phototherapy temperature required for this collaborative antibacterial platform is as low as 45 °C, which reduces the risk of damage to healthy tissue. In addition to using combination therapy, some materials with variable solid–liquid phase properties can also achieve low-temperature PTT. Hao Fu et al. [54] designed a thermal-responsive hydrogel (MeO-TSI@F127 NPs) with a low critical solution temperature (LCST). When the local temperature reached LCST (45 °C), the hydrogel automatically started the phase transformation process from liquid to solid, and light absorption was converted into the reflection of NIR light, such that the local temperature remained constant at 45 °C, thereby realizing low-temperature PTT.

**Figure 9:**
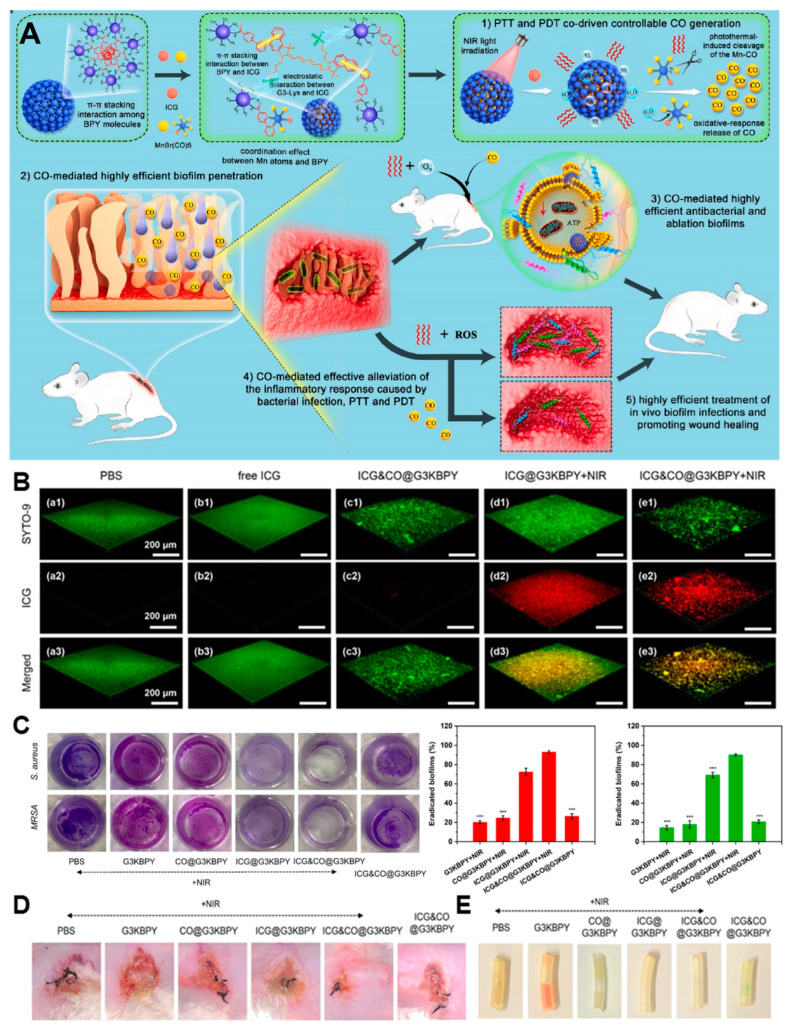
Antibacterial mechanism of ICG&CO@G3KBPY and antibacterial results in vivo and in vitro. (**A**) Schematic preparation of ICG&CO@G3KBPY for CO/PDT/PTT combating biofilm infection. (**B**) The penetration effect of ICG&CO@G3KBPY on *S. aureus* biofilm under NIR irradiation. (**C**) Crystal violet quantitative results of ICG&CO@G3KBPY in the in vitro eradication of biofilms. (**D**) Wound healing images. (**E**) Images of implants with bacteria remaining. Reprinted with permission from Ref. [55]. Copyright 2021 Elsevier Ltd.

Aside from the NO treatment, CO not only has a broad spectrum of antibacterial activities, but also can reduce the inflammation related to bacterial infection, with great application prospects in the antibacterial and antibiofilm fields. Xiaojun Cai and colleagues [55] developed a multi-mode collaborative antibacterial platform (ICG&CO@G3KBPY) for fighting biofilm infection driven by PDT and PTT. The nanoplatform is made by physically encapsulating the photosensitizer ICG and MnBr (CO)_5_ (CO precursor) into a nanogel (G3KBPY) based on a peptide dendrimer. Under irradiation with NIR light, the ICG in ICG&CO@G3KBPY can produce a large amount of ROS, and the ROS oxidize MnBr (CO)_5_ to generate CO. Meanwhile, the local temperature caused by the photothermic agent promotes the generation of CO, thus realizing the synergistic antibacterial effect of CO/PDT/PTT (Figure 9A). Under NIR irradiation (1 W·cm^−2^, 5 min), ICG&CO@G3KBPY (>150 μg·mL^−1^) treatment significantly reduced the level of ATP synthesis in bacteria, and the bactericidal rate against MRSA reached almost 100%. In in vitro biofilm eradication experiments, the biofilm permeability of the ICG&CO@G3KBPY treatment group reached up to 99% under NIR irradiation (Figure 9B), and the biofilm eradication rate was 93%, which is much higher than the 24% in the CO@G3KBPY treatment group and the 72% in the ICG@G3KBPY treatment group (Figure 9C). These results indicate that the synergistic effect of CO/PDT/PTT has excellent antibacterial and antibiofilm properties. In the medical catheter model and subcutaneous abscess model of mice infected with *S. aureus*, the ICG&CO@G3KBPY/NIR group can eradicate almost all biofilms on the catheter, and reduce the level of inflammatory factors and inflammatory cell infiltration, with rapid wound healing (Figure 9D,E). Moreover, ICG&CO@G3KBPY has excellent biocompatibility and anti-inflammatory activities which means it can effectively relieve the inflammatory response caused by PTT and PDT. CO/PDT/PTT’s synergistic eradication of biofilm is a safe and effective antimicrobial strategy.

In short, researchers continue to explore and develop a variety of organic nanocomposites through covalent and non-covalent interactions, and as far as possible to apply them in the field of antibiofilms. Antibacterial nanomaterials are modified or functionalized to combine them with antibiotics/PDT/PTT/gas therapy modes, which have great application prospects in the field of antibiofilm.

### 2.2. Construction of Organic Nanocomposites Formed by Covalent Interaction

Through covalent interactions, multi-segment functional molecules can be connected, such as photosensitizers, photothermic agents and other molecular components, which have stronger binding forces and higher stability. The reasonable design of covalent molecules is also carried out to improve the hardness, stiffness and plasticity properties of molecules [56], so as to achieve efficient antibacterial and antibiofilm effects.

**Figure 10:**
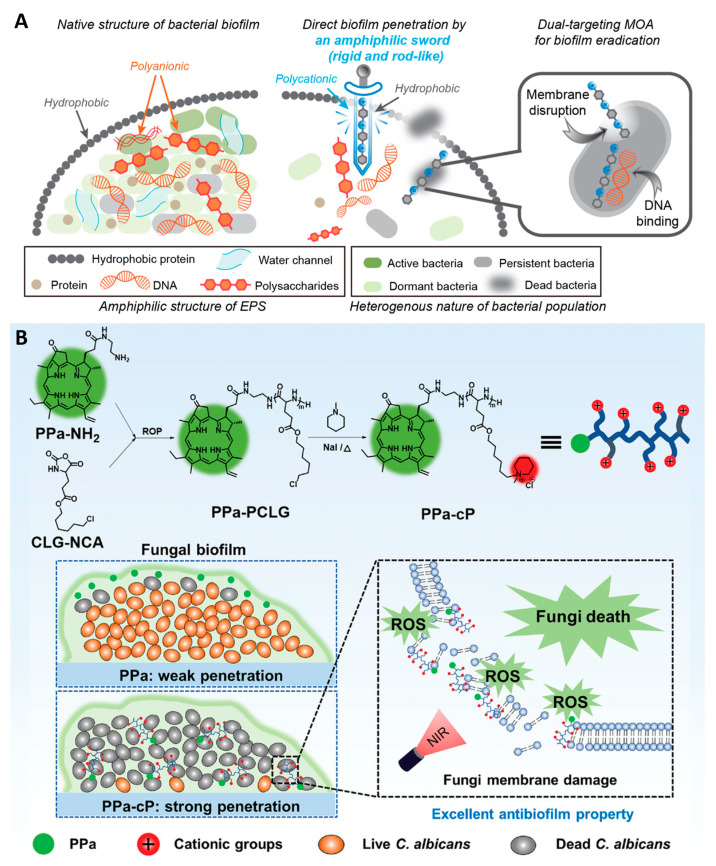
(**A**) Schematic diagram of antibiofilm mechanism of 3a oligomer and cationic polypeptide conjugate. Reprinted with permission from Ref. [57]. Copyright 2023 American Chemical Society. (**B**) Illustrated preparation of PPa-cP and mechanism diagram of killing *C. albicans*. Reprinted with permission from Ref. [58]. Copyright 2022 Wiley-VCH GmbH.

Xinxin Feng and colleagues [57] constructed an amphiphilic oligamine (3a) with a rigid structure for the direct penetration and eradication of biofilms via oligamine-based peptidomimetic studies (Figure 10A). Among the synthesized aggregates, 3a oligomer had the lowest MIC value (0.25–2 μg·mL^−1^) and MBIC value (16 μg·mL^−1^), and a strong broad-spectrum antibacterial activity against *S. aureus*. 3a oligomer was effective against dormant *S. aureus* (MIC = 0.5 μg·mL^−1^) with persistence (32 μg·mL^−1^ of 3a could completely kill bacteria after treatment for 30 min). The fluorescence colocalization of the 3a oligomer, cell membrane and DNA showed that the target of 3a was the DNA and bacterial membrane, and ROS was produced in this process. Therefore, membrane penetration, NDA destruction, and ROS oxidative stress were synergistic in antibacterial activity (Figure 10A). In vitro experiments on a blood infection model showed that the 3a oligomer could eradicate the *S. aureus* biofilm in blood without causing hemolysis. In vitro experiments of fibroblast biofilm infection models showed that 3a could completely eliminate all bacteria on the cell surface and restore the fibroblast to its normal morphology. Further, the 3a oligomer is covalently grafted onto PCL (polycaprolactone) to form a PCL-3A film, which has increased hydrophilicity and can effectively clear plankton bacteria to completely inhibit the formation of surface bacterial biofilms with broad-spectrum antibacterial and antibiofilm activities. In vivo wound treatment model experiments showed that the 3a oligomer can inhibit bacterial growth and reproduction, and promote rapid wound healing with excellent biosafety. In conclusion, the 3a oligomer with an amphiphilic rigid structure and membrane/DNA double antibacterial mechanism provides a new approach to the development of biofilm drugs and new medical devices.

To improve the antifungal effect, Pengqi Wan et al. [58] designed a pyrophosphoramide (PPa) covalently modified cationic polypeptide (cP) conjugate (PPA-cP) for the eradication of *C. albicans* biofilm. The cationic PPa-cP changes the permeability of the fungal membrane and promotes the effective penetration of PPa-cP by incorporating fungal recognition through electrostatic interaction. Under visible light irradiation, the photosensitizer PPa generates ROS and causes irreversible damage to the fungi (Figure 10B). The MIC values of PA-CP1 against *C. albicans* under light conditions were 1 × 10^−6^ M, showing strong antifungal activity. In in vitro biofilm experiments, PPa-cP can rapidly penetrate the biofilm of *C. albicans*, and at 20 × 10^−6^ M, 87.2% of the fungal biofilm can be removed with a fungal activity reduction inside the membrane to less than 20%. In a mouse wound infection model, the PPa-cP/light treatment group was able to clear 99.8% of the fungal colonies, and the hair follicles at the wound site were healthy, with fewer inflammatory cells. PPa-cP1 may provide a reference organic nanocomposite for the treatment of fungal biofilm infection in clinic. In addition, the scope of applications of PPa-cP1 in bacterial biofilm infection can be expanded.

## 3. Inorganic Nanomaterials

Compared with organic nanocomposites, inorganic nanomaterials have high stability, large specific surface areas, adjustable structures and sizes, and unique physical and chemical properties, giving them great application prospects in clearing biofilms. Traditional gold and silver antibacterial materials mainly achieve bactericidal effect through the release of metal ions [59], which can be made into nanoparticles to effectively reduce the leakage of metal ions, and can also give the material photothermal, photocatalytic, and photodynamic activities, thus greatly improving the antibiofilm efficiency of the material. In this section, metal-based nanomaterials are classified, and the applications of inorganic nanomaterials in antibiofilms based on gold, silver and other metals are described in detail.

### 3.1. Gold Based Inorganic Nanomaterials

Due to the surface plasmonic resonance (SPR) effect, Au can not only strongly absorb light in the NIR region, showing excellent photothermal properties, but can also increase the absorption spectrum range after modifying other metal oxides, and improve the photocatalytic ability so as to further enhance the photothermal capability. These properties mean Au NPs are widely used in antimicrobial and antibiofilm applications.

**Figure 11:**
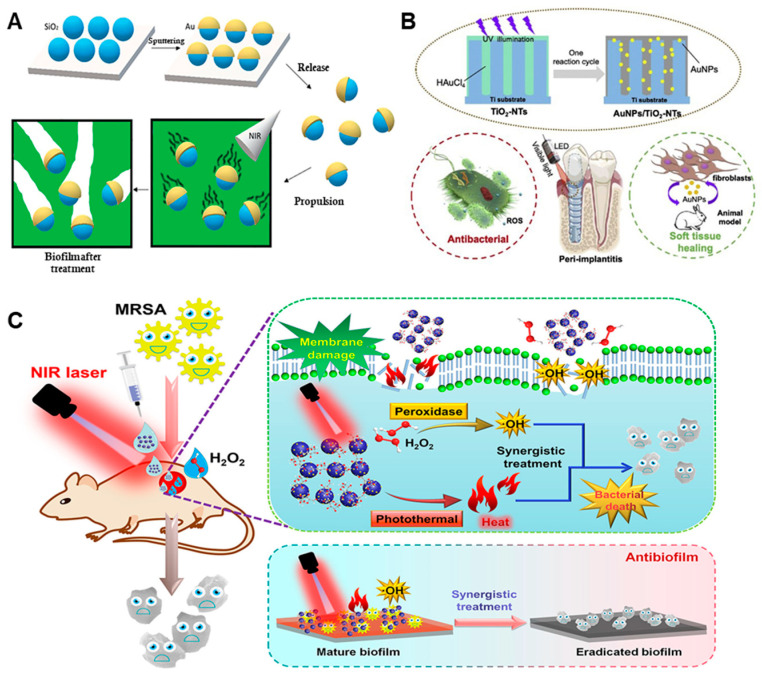
Schematic diagram of the antibacterial mechanism of three gold-based inorganic nanomaterials. (**A**) Illustrated preparation of mesoporous SiO_2_/Au nanomotor for dispersing biofilm under NIR. Reprinted with permission from Ref. [60]. Copyright 2023 John Wiley GmbH. (**B**) Schematic diagram of AuNPs/TiO_2_-NTs for eradicating biofilm, alleviating periodontal inflammation and promoting soft tissue healing. Reprinted with permission from Ref. [61]. Copyright 2020 Elsevier Ltd. (**C**) Illustrated mechanism of Au/MoO_3−x_ for bacterial biofilm eradication. Reprinted with permission from Ref. [62]. Copyright 2022 American Chemical Society.

Tijana Maric et al. [60] designed NIR light-driven self-propelled mesoporous SiO_2_/Au nanomotors for the eradication of *P. aeruginosa* biofilm. Under the irradiation of NIR light, Au in the mesoporous SiO_2_/Au nanomotor converts the absorbed light energy into heat energy to heat the nanomotor, thereby forming an asymmetric temperature gradient around the motor to make the nanomotor move in the direction of the low-temperature area, thus enabling the penetration and dispersal of the dense biofilm in situ (Figure 11A). The speed of motion of the mesoporous SiO_2_/Au nanomotor is positively correlated with the laser power. The biofilm eradication rate of the nanomotor when used against *P. aeruginosa* increased from 20% to 71% with the increase in illumination time from 30 s to 3 min. In addition, the high drug loading capability of the mesoporous SiO_2_/Au nanomotor means it has great application potential in the antibacterial field.

In addition to the photothermal properties of Au NPs, the plasmon resonance effect of Au NPs can improve the photocatalytic ability of photosensitizer materials to produce ROS. To further improve the eradication effect of antibiofilm agents, Xufeng Zheng et al. [61] reported a Au NPs-modified TiO_2_-NTs implant for inhibiting pathogens and promoting soft tissue healing. Under visible light irradiation, the incorporation of Au NPs can not only stimulate the rapid recombination of electrons and holes in TiO_2_, but also increases the rate constant to promote the generation of ROS (Figure 11B). The ROS production of Au NPs/TiO_2_-NTs increased with the increase in Au content. In in vitro antibiofilm experiments, the Au NPs/TiO_2_-NTs inhibition rate against multiple species of biofilm reached 99%. Even in the absence of light, the incorporation of Au NPs can significantly enhance the adhesion, migration and proliferation of fibroblasts on the surface of TiO_2_-NTs to effectively alleviate the inflammation of the tissue around the implant and promote the rapid healing of soft tissue. This work provides material for combating the inflammation caused by implants and improving the long-term performance of implants.

The plasmon properties of gold can not only improve the enzyme-like catalytic properties of nano-enzyme materials, but also enhance the photothermal properties. The combination of the enzyme-like activity and PTT associated with the Au-based materials can further augment the antibiofilm effect. Mengyu Cao et al. [62] designed an Au/MoO_3−x_ nanozyme with POD-like activity and photothermal properties for use in anti-MRSA therapy. The *K*_m_ value of the Au/MoO_3−x_ nanozyme is 3.1 times that of horseradish peroxidase (HRP), which has good POD-like activity and can catalyze H_2_O_2_ to generate ·OH, and the photothermal properties further improve its antibacterial and antibiofilm effects (Figure 11C). In in vitro antibacterial experiments, under NIR irradiation, the bactericidal rate of the Au/MoO_3−x_/H_2_O_2_ treatment group against MRSA reached 99.76%. In in vitro antibiofilm experiments, the biofilm formed by MRSA was almost eradicated. In in vivo antibacterial experiments, the collaborative treatment strategy effectively cleared the infected bacteria, and significantly promoted the epithelialization of wound tissue cells for the rapid healing of the wound, with high biocompatibility.

### 3.2. Silver-Based Inorganic Nanomaterials

As a type of broad-spectrum antibacterial material, the inherent toxicity of Ag NPs can induce bacterial oxidative stress, and the large amount of ROS produced will have toxic effects on bacteria. They will interact with the sulfur protein on the bacterial membrane to destroy the integrity of the cell membrane, leading to bacterial death. Ag NPs can also induce bacterial death by affecting ATP synthesis and blocking DNA replication. In addition, the SPR effect of Ag NPs gives them excellent photothermal properties [63,64]. Therefore, silver-based inorganic materials are widely used in the antibacterial and antibiofilm fields, such as in silver nanorings [65] and silver nanowire [66]. In addition, the development of new silver materials with antibacterial properties is also one development direction, such as silver peroxide and silver composite materials. Silver peroxide nanoparticles have unique biological effects and good photocatalytic activities, which can improve the oxidative stress response of microorganisms and achieve efficient sterilization. The multifunctional integration of a silver composite material can help realize multi-modal collaborative antibacterial effects.

**Figure 12:**
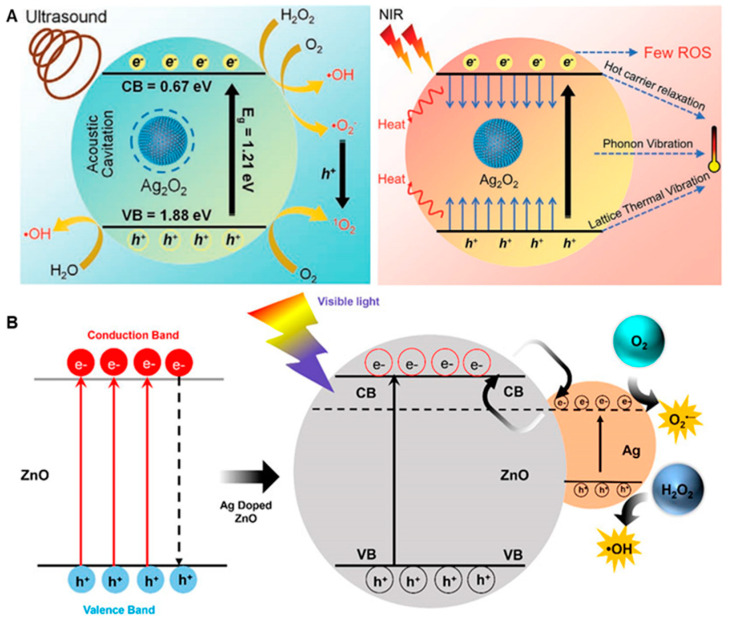
Schematic diagram of the antibacterial mechanism of silver-based inorganic nanomaterials. (**A**) Illustrated SDT and PTT of Ag_2_O_2_ NPs under US and NIR. Reprinted with permission from Ref. [67]. Copyright 2021 Wiley-VCH GmbH. (**B**) Schematic diagram of electron motion in ZnO@Ag and ROS generation under visible light irradiation. Reprinted with from Ref. [68].

Zhiling Zhu et al. [67] designed Ag_2_O_2_ NPs modulated by US and NIR light for antimicrobial and antibiofilm use. Ag_2_O_2_ NPs are synthesized at room temperature via a one-pot reaction of 1:1:1 in aqueous solution containing the protective stabilizers silver nitrate, sodium hydroxide and hydrogen peroxide. Under the irradiation of US and NIR light, Ag_2_O_2_ NPs promote the production of ROS in large quantities through the cavitation of microbubbles and the generation of electron–hole pairs for the purpose of killing bacteria, and their inherent toxicity will further improve the antibacterial activity, demonstrating the Ag/SDT/PTT synergistic antibacterial action of Ag_2_O_2_ NPs (Figure 12A). In in vitro antibacterial experiments, under US or NIR light irradiation, the bactericidal rate of the Ag_2_O_2_ NPs treatment group against *E. coli* and *S. aureus* was greater than 99.99%. Furthermore, Ag_2_O_2_ NPs can remove about 95% of MRSA biofilm. These results indicate that Ag_2_O_2_ NPs have excellent bactericidal and biofilm removal abilities. In the wound healing model of mice infected with MRSA, gel containing Ag_2_O_2_ NPs was applied to the wound. Under the irradiation of US and NIR, this effectively killed the infected bacteria at the wound and accelerated wound healing, with good biocompatibility. This work provides a new approach to designing metal peroxide nanomedicines with stimulus-response characteristics.

Ag@Ag_2_O core–shell nanocomposites (NCs) [69] showed good broad-spectrum bactericidal, antibiofilm and algicidal abilities, and Ag@Ag_2_O NCs showed significant antifungal activity at 50 μg·mL^−1^. However, the effective safety concentration of the nanomaterial is about 36.31 ± 1.53 μg·mL^−1^, which needs to be improved. Furthermore, Enoch Obeng et al. [68] reported a ZnO@Ag composite with photothermal/photodynamic effects that could be used for the eradication of *S. aureus* biofilms. ZnO@Ag nanocomposites are synthesized with the addition of different proportions of Ag NPs (0%, 0.5%, 2%, 8%). The combination of Ag promoted the generation of ROS in ZnO@Ag, which showed significant antibacterial and antibiofilm activities with the aid of a photothermal effect (Figure 12B). Compared with other nanocomposites, ZnO@8%Ag showed better photothermal and photodynamic properties. In in vitro antibacterial experiments, under NIR (2 W·cm^−2^, 10 min) light irradiation, the bactericidal rate of ZnO@8%Ag against *S. aureus* was more than 95%, which is much higher than that of ZnO. In in vitro biofilm inhibition experiments, biofilm formation was almost completely destroyed by ZnO@8%Ag under NIR (2 W·cm^−2^, 10 min) irradiation. These results indicate that ZnO@8%Ag has the excellent synergistic antibacterial activity of Ag/PDT/PTT. Importantly, ZnO@8%Ag has low hemolyticity and cytotoxicity. In in vivo antibacterial experiments, the ZnO@8%Ag/NIR treatment group effectively removed bacteria from wounds and promoted wound healing without causing obvious abnormalities in the body weight and major organs of the mice. In conclusion, ZnO@8%Ag nanocomposites have great application prospects in antibiofilms and wound healing.

### 3.3. Inorganic Nanomaterials Based on Other Metals

In addition to inorganic nanomaterials based on gold and silver precious metals, other precious metal nanomaterials such as platinum (Pt), palladium (Pd) and rubidium (Ru) have good catalytic activities and stability due to their unique electronic structure and SPR effect; they can catalyze the rapid generation of ROS and have been reported to have excellent antibacterial activities [70,71,72]. Further, the transition metal oxides involved in copper (Cu), zinc (Zn), titanium (Ti) and cerium (Ce) can be used as antibacterial agents in the treatment of bacterial infections due to their advantages of strong REDOX properties, non-toxicity, long-term stability and low cost [73,74,75,76,77].

The 2D PdCu alloy nanodendrites with POD-like activity designed by Guotao Yuan et al. [78] can be used to eliminate bacterial biofilms. The POD-like activity of the alloy was adjusted by accurately introducing Cu to regulate the electrostatic and dissociative adsorption of H_2_O_2_ on the surface of the alloy. Therefore, the POD-like activity of PdCu alloy nanodendrites is much higher than that of single-metal Pd. After reaching the biofilm, the PdCu alloy nanodendrites catalyzed the production of hydroxyl radicals to eliminate the bacterial biofilm with an eradication rate of 60%. Additionally, in order to further improve the antibiofilm activity of the material, Yixing Li and colleagues [79] synthesized FeNiTiCrMnCu_x_ high-entropy alloy nanoparticles (HEA-NPs) with excellent photothermal properties with the help of a high entropy effect, which was used to improve their antibacterial and antibiofilm performance. High-entropy alloys with different copper contents (x = 0, 0.25, 0.5, 1.0, 1.5 and 2.0) were prepared via the non-equilibrium arc discharge plasma method. Due to the presence of d-d interband transition, these alloys have good light absorption and photothermal conversion performance in the whole solar spectrum (250~2500 nm), and their average light absorption rate can reach 85%, which is conducive to photothermal sterilization. In in vitro antibacterial experiments, the inherent toxicity of Cu itself in alloys can effectively kill plankton bacteria, and the bactericidal rate increases gradually with the increase in Cu content. In in vitro antibiofilm experiments, 400 μg·mL^−1^ of FeNiTiCrMnCu_1.0_ could be used to remove 81% of *P. aeruginosa* and 96% of *B. vietnamensis* biofilms, and the clearance rate on *P. aeruginosa* biofilm increased to 97.4% under sunlight irradiation. Moreover, the high-entropy alloy can inhibit the synthesis of ATP in algae, thus inhibiting the growth and reproduction of algae, so it achieves a strong antibacterial performance in a natural environment. No matter whether it is illuminated or not, its antibacterial activity remains above 80% after five cycles of antibacterial experiments, indicating good recycling performance. In conclusion, the integration of antibacterial elements and non-contact heating property provides a feasible strategy for designing antibacterial materials with photothermal properties.

## 4. Organic/Inorganic Hybrid Nanomaterials

By modifying inorganic nanomaterials with organic nanomaterials, the surface charge of inorganic nanomaterials can be changed, and their targeting toward bacteria can be improved to realize the effective penetration of drugs into the biofilm. A cascade catalytic system can also be constructed by modifying related enzymes to achieve high antibacterial and antibiofilm effects. In addition, organic materials such as antimicrobial agents, precursors of antibacterial gas molecules and chiral molecules could be modified on inorganic nanomaterials to achieve multi-mode collaborative antibacterial utilization. This section classifies the functions of various components of hybrid materials, and mainly describes the research status of organic/inorganic hybrid nanomaterials (binary and ternary hybrid nanomaterials) used in antibiofilms in the past three years.

### 4.1. Binary Hybrid Nanomaterials

#### 4.1.1. Hybrid Materials Based on Magnetic Nanoparticles

Magnetic nanoparticles (MNP) are a class of magnetic nanomaterials with an Fe base (Fe_3_O_4_) as the main metal component, and have a wide range of applications in magnetic resonance imaging (MRI), optical imaging, nuclear imaging and ultrasound imaging [80]. Under the action of a magnetic field, magnetic nanoparticles can penetrate into the biofilm and excavate an additional channel to achieve the destruction of highly dense biofilm structures. However, the targeting of magnetic nanoparticles to biofilms is low, and the surface functionalization of the MNP can not only improve drug targeting, but can also cause it to combine with PDT, PTT or antibiotics, thus realizing synergistic antibacterial effects.

**Figure 13:**
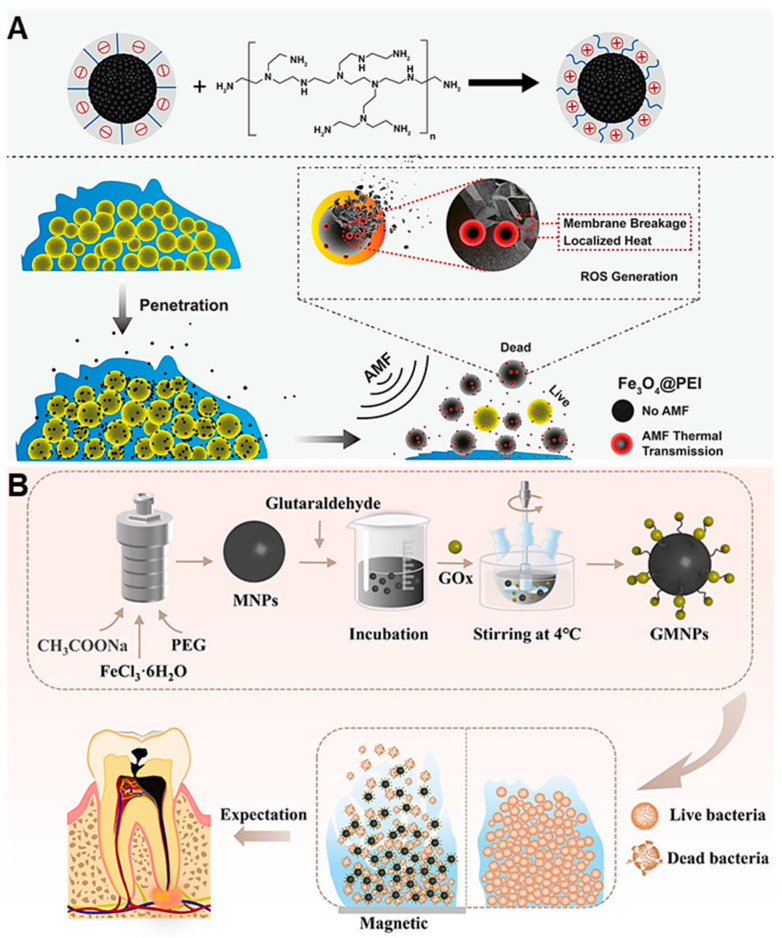
(**A**) Illustrated preparation of Fe_3_O_4_@PEI NPs for scavenging biofilm under an alternating magnetic field. Reprinted with permission from Ref. [81]. Copyright 2022 American Chemical Society. (**B**) Synthesis and preparation of GMNPs. Reprinted with permission from Ref. [82]. Copyright 2021 American Chemical Society.

Wenhui Liu and colleagues [81] designed a positively charged magnetic nanoparticle (Fe_3_O_4_@PEI NPs) for use in antimicrobial and antibiofilm therapy. Firstly, the negatively charged mesoporous Fe_3_O_4_NPs were synthesized by a solvothermal method, and then positively charged Fe_3_O_4_@PEI NPs were obtained through PEI functionalization on Fe_3_O_4_NPs (Figure 13A), which could be targeted to identify bacteria. Under an alternating magnetic field, the Fe_3_O_4_@PEI NPs underwent physical rotation to convert internal energy into heat energy, resulting in physical stress and thermal damage to the biofilm. After that, ROS were produced by bacterial oxidative stress, further enhancing the bactericidal and antibiofilm effects (Figure 13A). In in vitro antimicrobial experiments, 800 μg·mL^−1^ of Fe_3_O_4_@PEI NPs was shown to reduce *S. aureus* and *E. coli* by 4 and 5 orders of magnitude, respectively, under alternating magnetic fields. In the five antibacterial cycles, the bactericidal effect of Fe_3_O_4_@PEI NPs was basically consistent, implying a good recycling performance. In in vitro antibiofilm experiments, 800 μg·mL^−1^ of Fe_3_O_4_@PEI NPs was able to clear 87.4%, 84.9% and 85.8% of *S. aureus*, *E. coli* and *P. aeruginosa* biofilms, respectively, under an alternating magnetic field.

To further improve the antibiofilm efficiency of magnetic nanoparticles, Qihui Zhou and colleagues [82] designed and synthesized glucose oxidase (GOx)-modified magnetic nanoparticles (GMNPs) to eradicate microbial biofilms in the model of persistent endodontic infections (PEIs) in a cascade catalytic/physical damage manner. Fe_3_O_4_ NPs were synthesized via a REDOX reaction between ferric chloride and ethylene glycol, followed by modification with GOx to obtain GMNPs (Figure 13B). Under the action of a magnetic field, the moving GMNPs destroyed the three-dimensional structure of the biofilm to promote the effective penetration of ROS for the enhancement of bactericidal efficiency. GMNPs can produce ROS in two ways: (1) on the membrane surface, GOx catalyzes glucose to produce hydrogen peroxide, and MNPs further catalyze hydrogen peroxide to generate hydroxyl radical; (2) inside the membrane, on the basis of GOx catalyzing glucose to produce hydrogen peroxide, microbial oxidoreductase/MNPs participate in catalyzing hydrogen peroxide to produce free radicals. In vitro antimicrobial experiments showed that 1 mg·mL^−1^ of GMNPs could kill more than 80% of *E. faecalis* and 98.84% of *C. albicans*. In in vitro anti-biofilm experiments, GMNPs can significantly remove the biofilm formed by bacteria/fungi under the action of a magnetic field. GMNPs at 1 mg·mL^−1^ had a lower hemolysis rate and no obvious toxicity toward cells. GMNPs offer a new strategy for eradicating PEIs-associated bacteria/fungi.

#### 4.1.2. Hybrid Materials Based on Gold Nanomaterials

As a commonly used antibacterial material, the antibacterial activity of gold-based nanomaterials is also related to their morphology. At present, many gold-based nanostructures have been designed, including Au NPs, gold nanoclusters (Au NCs), gold nanobipyramids (Au NBPs), gold nanorods (Au NRs) and gold nanostars (Au NSs) [83]. Gold nanoparticles with different morphologies were used in combination with antibiotics, such as ciprofloxacin-conjugated Au NRs for eradicating MRSA biofilm [84] and Au NSs Plus Amikacin for inhibiting carbapenem-resistant *K. pneumoniae* biofilm [85]. In addition, the surface functionalization of the gold nanomaterials can improve the targeting of bacteria and realize multi-mode antibacterial effects.

**Figure 14:**
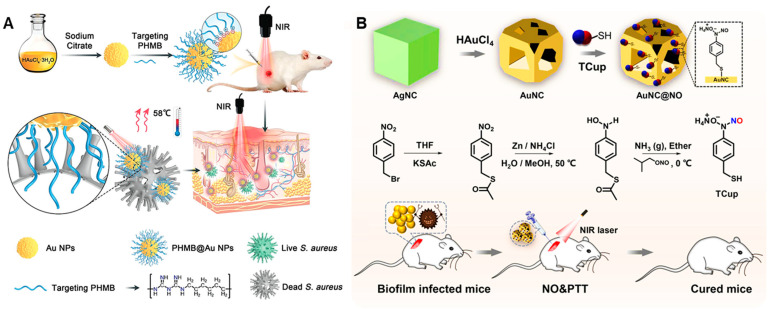
(**A**) Illustrateion of the preparation of PHMB@Au NPs for anti-infection treatment in vivo. Reprinted with permission from Ref. [86]. Copyright 2022 the authors. (**B**) Schematic preparation of AuNC@NO for collaborative NO/PTT in MRSA biofilm-infected wound model. Reprinted with permission from Ref. [87]. Copyright 2021 American Chemical Society.

Xiaojun He and colleagues [86] developed a photothermal platform (PHMB@Au NPs) consisting of polyhexamethylbiguanide (PHMB) and Au NPs via electrostatic interaction with bactericidal and antibiofilm functions for the treatment of wound infections caused by *S. aureus*. As an antibacterial agent, PHMB can easily adhere to and effectively kill bacteria. Positively charged nanoparticles can target and identify bacteria for their effective penetration. Under NIR irradiation, the photothermal properties of Au NPs can increase the temperature of the infected site and realize photothermal sterilization (Figure 14A). In in vitro antibacterial experiments, the bactericidal rate of PHMB@Au NPs at 9 μg·mL^−1^ against *S. aureus* was above 95% under irradiation with NIR (2.0 W·cm^−2^, 5 min). In in vitro biofilm experiments, PHMB@Au NPs can inhibit the formation of biofilm up to 85%, and strongly restrain the secretion of hemolysin from *S. aureus*. In subcutaneous infection and abscess models, PHMB@Au NPs can quickly remove bacteria from the wound site and significantly reduce inflammation and abscesses. In addition, PHMB@Au NPs are almost non-toxic to cells under NIR irradiation, exhibiting good biocompatibility. The use of the chemotherapeutic/PDT photothermal system is a potential alternative to antibiotic therapy.

Yizhang Tang and colleagues [87] designed an antimicrobial nanoplatform AuNC@NO that releases NO in response to NIR and enhances the efficacy of PTT for biofilm therapy (Figure 14B). First, gold nanocages (AuNCs) were prepared by the galvanic replacement reaction of AgNC and HAuCl_4_. The temperature-responsive NO donor TCup was fixed on AuNCs to obtain AuNC@NO via the thiol–gold interaction, which can continuously release NO at normal physiological temperature. Under NIR irradiation, the photothermal effect of AuNC@NO can inactivate bacteria, and the release of NO and Ag+ is accelerated by the increase in local temperature, inducing bacteria to produce ROS under oxidative stress, thus causing irreversible damage to bacteria. In in vitro antibacterial experiments, under NIR (0.5 W·cm^−2^, 5 min) irradiation, AuNC@NO can reduce the number of MRSA by 4 orders of magnitude. In in vitro biofilm removal experiments, 100 pM of AuNC@NO effectively dispersed the formed biofilm, and the residual bacteria in the membrane decreased by 6 orders of magnitude. These results indicate that AuNC@NO has good antibacterial and antibiofilm activity. In the in vivo implant biofilm infection model, under the irradiation of NIR (0.5 W·cm^−2^, 5 min), 100 pM of AuNC@NO significantly cleared the biofilm on the implant, and its bactericidal rate on the infected site of the wound was 94.4%, facilitating rapid wound healing. At the concentration of 100 pM, its hemolytic and cell viability were lower than 5% and higher than 80%, respectively, demonstrating good biocompatibility. Therefore, AuNC@NO nanocomposites have broad application prospects in the treatment of health care-associated infections.

**Figure 15:**
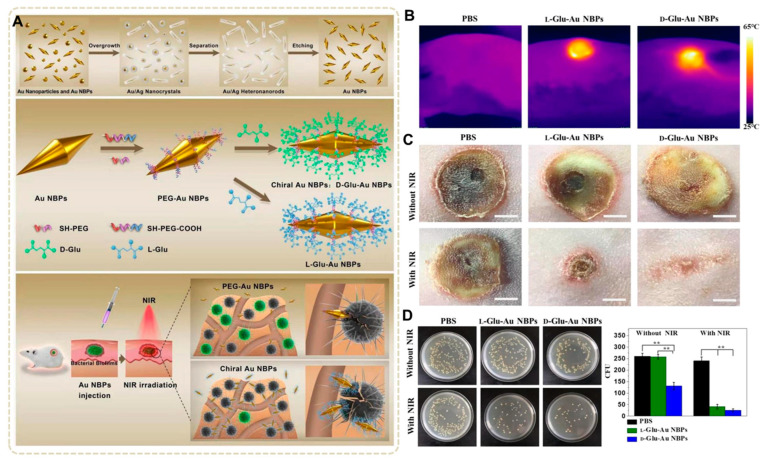
Synthesis and in vivo antibiofilm activity of gold nanobipyramids (Au NBPs) hybrid materials. (**A**) Schematic synthesis of D/L-Glu-Au NBPs for in vivo biofilm clearance. (**B**) Thermal imaging of D/L-Glu-Au NBPs in mice under NIR irradiation. (**C**) Pictures of wound infected with *S. epidermidis* after different treatments. (**D**) CFU counting plate pictures and quantitative results of residual bacteria in wound infection. Reprinted with permission from Ref. [88]. Copyright 2020 Elsevier Ltd.

In order to improve the targeting of the antibiofilm, Min Zhang et al. [88] constructed chiral glutamate-functionalized Au NBPs for combating bacterial and biofilm infections. Au NBPs were synthesized by etching, and then modified with D-glutamic acid (D-Glu) or L-glutamic acid (L-Glu) to form D/L-Glu-Au NBPs (Figure 15A). The glutamic acid in D/L-Glu-Au NBPs can enhance the binding effect on the bacterial cell wall, and the two tips of the Au NBPs promote the effective penetration of D/L-Glu-Au NBPs into bacteria for the enhancement of the PTT effect under NIR light irradiation. In addition, D-Glu can inhibit the catalytic steps associated with D-Glu to disrupt the biosynthesis of peptidoglycan, thereby causing severe damage to bacteria, and the mechanism of action is similar to that of antibiotics. In in vitro antibacterial experiments, the bactericidal rate of D-Glu-Au NBPs against *S. epidermidis* in the absence of NIR was up to 70%, while L-Glu-Au NBPs and D-Glu-Au NBPs under NIR light could kill 85% and 95% of bacteria, respectively. In in vitro antibiofilm experiments, L-Glu-Au NBPs and D-Glu-Au NBPs can remove about 80% and 90% of bacterial biofilms under NIR light irradiation. These results indicate that chiral Au NBPs have good antibacterial and antibiofilm properties when used in synergistic chemotherapy, as well as PTT and physical effects. In the internal wound treatment, the photothermal effect induced by the D/L-Glu-Au NBPs treatment group was mainly concentrated in the infected region (Figure 15B), which could help it to significantly clear the infecting bacteria at the wound site (Figure 15D) without obvious thermal damage to healthy tissue, thus promoting rapid wound healing (Figure 15C). In summary, the chiral surface modification technique of nanomaterials provides a promising method for improving the bactericidal effect of PTT.

### 4.2. Ternary Hybrid Nanomaterials

**Figure 16:**
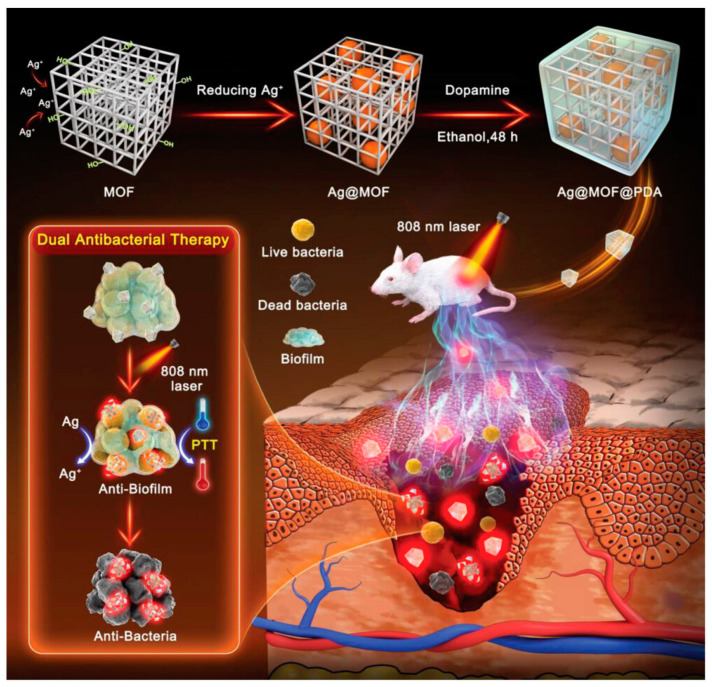
Illustrated synthesis of Ag@MOF@PDA for antibacterial and antibiofilm treatment. Reprinted with permission from Ref. [90]. Copyright 2023 Wiley-VCH GmbH.

MOF materials are self-assembled from metal and organic ligands through coordination, and have been widely used in separation, catalysis, sensing and biomedicine due to their good biocompatibility and easy regulation [89]. Yaping He and colleagues [90] built a photothermal antibacterial nanoplatform (Ag@MOF@PDA) for the treatment of bacterial and biofilm infections (Figure 16). Ultrafine Ag NPs (Ag@MOF) were synthesized in cyclodextrin metal–organic framework crystals via the diffusion method, and then Ag@MOF was encapsulated in an in situ PDA shell to produce Ag@MOF@PDA, whereby Ag^+^ undergoes long-term and controllable release. The protective effect of PDA on Ag^+^ reduces the release rate of Ag^+^, which in turn increases the release time of Ag^+^. Meanwhile, the increase of local temperature can effectively kill bacteria and significantly promote the release of Ag^+^. Ag^+^ induces oxidative stress in bacteria to produce ROS to cause severe damage to bacteria (Figure 16). In in vitro antibacterial and antibiofilm experiments, Ag@MOF@PDA can kill more than 95% of bacteria and remove 76% of bacterial biofilm under NIR irradiation. In an in vivo infection model, Ag@MOF@PDA/NIR virtually killed the bacteria at the site of infection and eradicated the biofilm, reducing inflammatory cell infiltration and promoting rapid wound healing with good biocompatibility.

In order to improve the antibiofilm efficiency and targeting of MOF materials, Xin Fan et al. [91] designed a nano-catalyst (Ni@Co-NC) with a nanohook structure and POD-like activity for eradicating bacterial biofilms. After reaching the biofilm, the nano-hook automatically connected the bacteria and the biofilm, which significantly improved the binding ability of the catalyst to bacteria. Then, the nano-enzyme catalyzed H_2_O_2_ to generate toxic hydroxyl radicals. Under NIR irradiation, the nano-catalyst can cause local temperature increases, realizing the synergistic antibiofilm effect of PDT/PTT. Under NIR irradiation, the bactericidal rate against MRSA in the Ni@Co-NC/H_2_O_2_ group was 89.95%, which is much higher than that of Ni@ZIF67-C without a nano-hook structure. Ni@Co-NC/H_2_O_2_ cleared 67.74% of the biofilm in the dark, while its biofilm clearance increased to almost 100% under NIR irradiation. In the wound infection model, the Ni@Co-NC/H_2_O_2_ treatment group eradicated bacteria at the wound site, and accelerated the rapid healing of the wound. When 62.5 μg·mL^−1^ of Ni@Co-NC was co-incubated with human keratinocytes, its cell viability was still above 90%, showing excellent biocompatibility. However, due to its unknown cumulative toxicity, there are potential pitfalls related to intravenous administration and the treatment of diseases caused by unknown bacteria.

**Figure 17:**
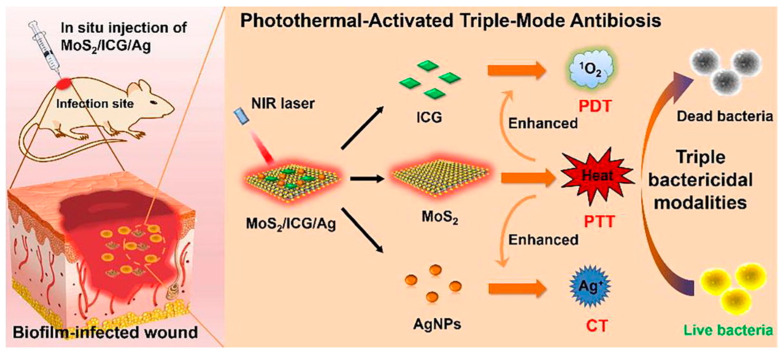
Schematic diagram of the photothermally activated three-mode collaborative antibacterial nanoplatform (MoS_2_/ICG/Ag) for eradicating bacterial biofilm. Reprinted with permission from Ref. [95]. Copyright 2021 Elsevier Ltd.

As a non-precious metal element, molybdenum oxide series [92], molybdenum sulfide series [93] and molybdenum carbide series [94] have more applications in the fields of photocatalysis, optoelectronics and antibiofilm due to their excellent physical and chemical properties and photothermal properties. In recent years, multi-mode collaborative antibacterial molybdenum-based materials have been reported to further improve the eradication effect of biofilm. Huan Li and colleagues [95] constructed a chemical/photothermal/photodynamic antibacterial nanoplatform (MoS_2_/ICG/Ag) with three modes (Figure 17). Under NIR irradiation, MoS_2_ nanosheets can generate heat and increase the local temperature, thus accelerating the release of ICG and Ag^+^ to produce a large amount of ROS and induce physical damage to the bacteria. Meanwhile, the load of ICG and Ag^+^ on the nanosheets will in turn enhance the photothermal effect and achieve the result of mutual strengthening, thereby realizing the three-mode synergistic antibacterial effects (Figure 17). In in vitro antibacterial experiments, under NIR (1.0 W·cm^−2^, 10 min) light irradiation, the bactericidal rate of MoS_2_/ICG/Ag at 150 μg·mL^−1^ against *S. aureus* was 99%, and the bactericidal rate of MoS_2_/ICG/Ag at 250 μg·mL^−1^ against *E. coli* was 99%. The results show that the nanoplatform was more likely to kill Gram-positive bacteria. In in vitro antibiofilm experiments, the MoS_2_/ICG/Ag group was able to inhibit 77.9% of biofilm formation and kill 96.8% of bacteria in the mature biofilm under NIR irradiation. These results suggest that MoS_2_/ICG/Ag has an excellent synergistic effect in inhibiting biofilm formation and eradicating bacteria deep within the biofilm. In in vivo antibacterial and antibiofilm experiments, MoS_2_/ICG/Ag treatment achieved low-temperature PTT at a local temperature of 46.6 °C, and cleared more than 98% of the bacteria at the wound site without serious inflammation. This work provides a theoretical basis for the design of multi-modal combined antibacterial agents.

**Figure 18:**
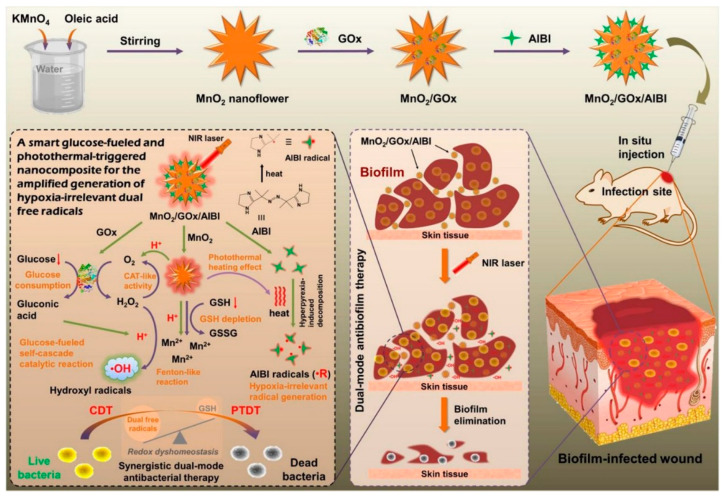
Schematic diagram of the synthesis and antibacterial mechanism of the biradical antibacterial nano-agent (MnO_2_/GOx/AIBI) triggered by light and heat. Reprinted with permission from Ref. [96]. Copyright 2022 Elsevier Ltd.

Because the biofilm is in a hypoxic state, ROS production will be limited. In order to increase the production of free radicals, in addition to supplementing oxygen to relieve hypoxia, it is important to design an antibacterial strategy that is not affected by oxygen content and can also produce a large number of toxic free radicals. Dinggeng He’s group [96] constructed a biradical antibacterial nanoplatform (MnO_2_/GOx/AIBI) that is not affected by oxygen content for the treatment of associated infections caused by bacterial biofilms. By loading GOx and a thermally active azo initiator (AIBI) on a flower-like manganese dioxide carrier, a photothermally triggered biradical antibacterial agent (MnO_2_/GOx/AIBI) was successfully constructed. The GOx in MnO_2_/GOx/AIBI catalyzes the conversion of glucose to gluconic acid and hydrogen peroxide. On the one hand, manganese dioxide with CAT enzyme activity continued to catalyze the decomposition of hydrogen peroxide to produce O_2_, which supplemented the consumption of O_2_ by GOx; on the other hand, excessive GSH was consumed and MnO_2_/GOx/AIBI decomposed into Mn^2+^ with POD-like activity, triggered by the acid and hydrogen peroxide, which catalyzed hydrogen peroxide to produce ·OH. At the same time, manganese dioxide also has good photothermal properties and photothermal stability. Under NIR irradiation, the local temperature increase caused by manganese dioxide can activate the decomposition of AIBI and produce alkyl free radicals (·R), which is not affected by oxygen content, so as to realize the photothermal dynamic therapy (PTDT) of bacterial biofilm (Figure 18). In in vitro antibacterial experiments, under the irradiation of NIR (1.0 W·cm^−2^), the bactericidal effect of MnO_2_/GOx/AIBI against *S. aureus* was 99%. In in vitro antibiofilm experiments, the group of MnO_2_/GOx/AIBI+glucose inhibited 64.7% of biofilm formation under NIR irradiation, and effectively killed 88.5% of *S. aureus* in the mature biofilm. These results indicate that MnO_2_/GOx/AIBI has significant antibacterial and antibiofilm properties in vitro. In in vivo antibiofilm experiments, after treatment with MnO_2_/GOx/AIBI under NIR irradiation, the local temperature at the infection site was increased to 42.3 °C, and the inflammatory response was reduced with rapid wound healing. There is almost no cytotoxicity and hemolysis to HEK 293 and red blood cells, demonstrating excellent biocompatibility. Thus, MnO_2_/GOx/AIBI is a supplementary strategy for the treatment of biofilms in a hypoxic environment.

## 5. Summary and Prospect

Microbial biofilms, as a metabolic and protective barrier, largely hinder the penetration of antimicrobials for further microbial killing, thus leading to the generation of multidrug-resistant pathogens to a large extent. In recent years, nanomaterials have been widely used in medical devices, food packaging, cosmetics and antibacterial fields. Among them, nanocomposites applied in the field of bacterial infection have a variety of antibacterial mechanisms against bacteria and their biofilms. In recent years, nanocomposites have shown great potential in inhibiting biofilm formation and eradicating biofilms by reducing bacterial and biofilm adhesion, increasing drug permeability and stability, allowing active targeting (charge surface modification, microenvironmental stimulus response), and collaborating with various therapeutic modes including PTT, PDT and SDT. In this review, the application of nanocomposites in the field of antibiofilms is summarized, and the antibiofilm effect and the possible antibacterial mechanism of organic nanocomposites, inorganic nanomaterials and organic/inorganic hybrid nanomaterials are introduced (Table 1). The specific treatment methods mainly include chemotherapy (antibiotics and metal ions in the composite), PTT (various photothermal agents and metal components in the composite), PDT (photosensitizer in the composite), physical damage (magnetic movement) and enzyme-like catalysis (POD). It should be noted that most of the composite nanomaterials mentioned are expected to alleviate the generation of multidrug-resistant bacteria and their biofilms, and have significant effects on wound healing and reducing implant infection, providing basic support for the clinical treatment of diseases caused by pathogenic bacteria and their biofilms.

Although nanocomposites can efficiently remove biofilms, there is little research on the molecular antibacterial mechanism of nanomaterials. Some studies have shown that certain molecules play a key role in various processes of biofilm formation, and if the designed nanomaterials can destroy key molecules, the formation of biofilm can be significantly inhibited [2]. Therefore, the design of targeted drugs to destroy proteins, polysaccharides or nucleic acids in EPS at the molecular level can significantly improve the eradication rate of biofilms. For example, DNase targeting the recognition of DNA on the matrix and protease K targeting the recognition of matrix proteins can decompose DNA and proteins, thereby dispersing biofilms [52,97], which is similar to the electrostatic recognition targeting of bacteria and drugs. The mechanism of targeted recognition of nanocomposites and active molecules needs to be further explored at the molecular level. In addition, if the nanocomposite is designed to act directly on the genes that control the synthesis of the key molecules, it may also have the effect of inhibiting biofilm formation.

At present, the research on nanocomposites is mostly in the experimental stage, in vitro and in vivo, and the established biofilm model is mostly based on a single strain. In the actual situation of bacterial infection, the problem of multiple biofilms arises, thus precluding the influence of multiple microbial interactions, which may lead to different experimental results in vivo and in vitro.

More importantly, the toxicity of nanomaterials has been widely addressed. Inorganic nanomaterials such as metals can easily cause oxidative stress and inflammation [98]. For example, nano-iron and nano-manganese in welding smoke may induce oxidative stress and inflammation, leading to lung damage [99]. Metal nanoparticles (Ag, ZnO, TiO_2_) are most likely to induce polycystic ovary syndrome, follicular atresia and inflammation [100]. If these metal nanoparticles accumulate in the human body for a long time, they may accumulate toxicity against internal organs and lead to organ lesions. In contrast, most organic nanocomposites have good biocompatibility [46,47,51,55]. Therefore, one way to adjust the biocompatibility of inorganic nanomaterials is by modifying organic nanomaterials with good biocompatibility to obtain organic/inorganic nanomaterials. However, current toxicity studies on organic nanomaterials are still not comprehensive enough. Whether they will affect life activities in the human body or interact with biological macromolecules, their mechanism of action and results are still unknown. It is important that the research in nanomaterials for use as antibiofilms mostly uses mice or rabbits as experimental models to study their biosafety in vivo. However, there is a big gap between them and the actual situation of the human body. The compatibility of cells, tissues and organs here cannot be compared to those inside the human body, and the theoretical data provided by the in vitro model do not accurately correspond to the overall state of adaptation of the human body. Moreover, nanocomposites should not only have excellent antibiofilm properties, but also have the advantages of rapid degradation and timely discharge after playing a role, so as to ensure biosafety to the human body.

Nanocomposites with high targeting, high degradability, low toxicity and efficient antibacterial and antibiofilm activities have broad application prospects in the future, and their potential properties are still to be developed. In summary, it is expected that this review can help readers understand the application status of composite nanomaterials in the field of antibiofilm in recent years, and provide useful ideas for the development and design of efficient and biosafe antibiofilm nano-agents.

## Figures and Tables

**Table 1:** Summary of nanocomposite materials for biofilm eradication.

Anti-Biological Film Agent	Functional Unit	Types of Anti-Biofilm	Mechanism	References
Pep@Ce6 micelle	Coupling photosensitizing agent Ce6 (α-CD-Ce6), PEG-Pep	Reduced the thickness of *P. aeruginosa* biofilm from 25 μM to 14 μM.	PDT	[45]
PECL@PTTA micelle	The hydrophilic fragment PECL-ad, hydrophobic fragment cd-PTTA includes β-cyclodextrin, PBA, photosensitizer TPE, thione linker and Amp	The eradication rate of MRSA biofilm was 83%.	Antibiotic Amp, PDT	[46]
Arg-CD-AcMH	L-arginine modified with β-CD (hydrophilic fragment), acetalized maltoheptaose modified with ferrocene (hydrophobic fragment), GOx and GA	The biofilm formed by *E. coli* and *S. aureus* could be almost completely eliminated.	Enzymatic cascade catalysis continuously releases NO and damages biofilm.	[47]
α-CD-Ce6-NO-DA	NO-prodrug (α-CD-NO), photosensitizer Ce6-prodrug (α-CD-Ce6) and pH-sensitive PEG-(KLAKLAK)_2_-DA	The MRSA biofilm could be almost completely eliminated.	NO/PDT	[1]
CGL-Alz NCs	Alizarin, liposome and chitosan–gum arabic-coated.	The formation rate of *C. albicans* and *E. coli* biofilm were 90–95%	Leading to protein inactivation, interaction with nucleic acids (DNA and RNA), alteration of efflux pumps, and destruction of membrane integrity.	[48]
Lip-Ce6-PFH@O_2_	O_2_ carrier PFH, photosensitizer Ce6 and liposome	The eradication rate of *P. aeruginosa* biofilm is 90.1%.	O_2_ enhanced PDT	[49]
RB@PMB@GA NPs	RB-NH_2_, PDA, PMB and gluconic acid	At pH 5.0, the penetration and eradication rate of *P. aeruginosa* biofilm were, respectively, 93% and 100%.	PDT/PTT synergistic antibacterial activity.	[50]
AZM-DA NPs	PAMAM-AZM NPs, PEG-b-PLys	The bactericidal rate of AZM-DA NPs for bacteria in *P. aeruginosa* biofilm was 99.998%.	Antibiotic AZM	[51]
PRZ nanocomplex	Protease K, photosensitizer RB and ZIF-8.	The eradication rate of *S. aureus* biofilm was 95%.	Protease K decomposes the protein, PDT	[52]
AI-MPDA	L-Arg, MPDA and photosensitizer ICG	The eradication rate of *S. aureus* biofilm was 99%.	NO/PDT/PTT	[53]
ICG&CO@G3KBPY	ICG, CO precursor MnBr(CO)_5_ and nanogel G3KBPY.	The penetration and eradication rate of *S. aureus* biofilm were, respectively, 99% and 93%.	CO/PDT/PTT	[55]
Amphiphilic oligamine(3a)	p-ethylphthalimidate and various diamines	MBIC (*S. aureus*, *E. coli*, *P. aeruginosa*) = 16 μg·mL^−1^, MBEC (*S. aureus*, *E.coli*, *P. aeruginosa*) = 64, 128 and 16 μg·mL^−1^.	Membrane penetration, DNA destruction, and ROS oxidative stress were synergistic in antibacterial activity.	[57]
PPa-cP	Pyrophosphoramide (PPa), cationic polypeptide (cP)	The eradication rate of *C. albicans* biofilm was 87.2%.	PDT	[58]
Mesoporous SiO_2_/Au nanomotors	Mesoporous SiO_2_, Au NPs	The eradication rate of *P. aeruginosa* biofilm was 71% at 3 min.	Physical damage	[60]
Au NPs/TiO_2_-NTs	Au NPs, TiO_2_-NTs	The inhibition rate of multispecies biofilms was 99%.	ROS	[61]
Au/MoO_3−x_ Nanoenzyme	MoO_3−x_, Au NPs	The MRSA biofilm could be almost completely eliminated.	POD-like/PTT	[62]
Ag_2_O_2_ NPs	Ag_2_O_2_ NPs	The eradication rate of MRSA biofilm was 95%.	Ag/SDT/PTT	[67]
ZnO@Ag nanocomposites	Different proportions of Ag NPs (0%, 0.5%, 2%, 8%), ZnO	The formation of *S. aureus* biofilm could be almost completely inhibited.	Ag/PDT/PTT	[68]
2D PdCu alloy nanodendrites	Cu, Pd	The eradication rate of *P. aeruginosa* biofilm was 60%.	POD-like	[78]
FeNiTiCrMnCu_x_ HEA-NPs	Cu	The eradication rate of *P. aeruginosa* biofilm was 97.4%.	PTT	[79]
Fe_3_O_4_@PEI NPs	Fe_3_O_4_ NPs, PEI	The eradication rates of *S. aureus*, *E. coli* and *P. aeruginosa* biofilms were, respectively, 87.4%, 84.9% and 85.8%.	Physical damage/PTT	[81]
GMNPs	GOx, magnetic nanoparticles	The *E. faecalis* and *C. albicans* biofilms could be almost completely eliminated.	Cascade catalysis/physical damage	[82]
PHMB@Au NPs	Antibacterial agent PHMB, Au NPs	The inhibition rate of *S. aureus* biofilm was 85%.	Chemotherapeutic/PDT	[86]
AuNC@NO	AuNC, NO donor Tcup	Dispersing MRSA biofilm, resulting in a decrease of 6 orders of magnitude in residual bacteria within the biofilm.	NO/PDT	[87]
D/L-Glu-Au NBPs	D-Glu, L-Glu and Au NBPs	The eradication rate of the *S. epidermidis* biofilm was, respectively, 80% and 90% by L-Glu-Au NBPs and D-Glu-Au NBPs.	PTT	[88]
Ag@MOF@PDA	CD-MOF, PDA and Ag NPs	The eradication rate of *S. aureus* biofilm was 75%.	Ag/PDT/PTT	[90]
Ni@Co-NC	MOF, Co/Ni NPs	The MRSA biofilms could be almost completely eliminated.	POD-like/PTT	[91]
MoS_2_/ICG/Ag	Ag NPs, MoS_2_ and ICG	The inhibition and eradication rates of *S. aureus* biofilm were, respectively, 77.9% and 96.8%.	chemical/PTT/PDT	[95]
MnO_2_/GOx/AIBI	MnO_2_,·R precursor AIBI and GOx	The inhibition and eradication rates of *S. aureus* biofilm were, respectively, 64.7% and 88.5%.	PDT/PTT related to hypoxia.	[96]

## Data Availability

Data sharing not applicable.

## References

[1] Hu D., Deng Y., Jia F., Jin Q., Ji J. (2020). Surface Charge Switchable Supramolecular Nanocarriers for Nitric Oxide Synergistic Photodynamic Eradication of Biofilms. ACS Nano.

[2] Li X., Chen D., Xie S. (2021). Current Progress and Prospects of Organic Nanoparticles against Bacterial Biofilm. Adv. Colloid Interface Sci..

[3] Abu Bakar M., McKimm J., Haque S.Z., Majumder M.A.A., Haque M. (2018). Chronic Tonsillitis and Biofilms: A Brief Overview of Treatment Modalities. J. Inflamm. Res..

[4] Niedzielski A., Chmielik L.P., Stankiewicz T. (2021). The Formation of Biofilm and Bacteriology in Otitis Media with Effusion in Children: A Prospective Cross-Sectional Study. Int. J. Environ. Res. Public Health.

[5] Lerche C.J., Schwartz F., Theut M., Fosbol E.L., Iversen K., Bundgaard H., Hoiby N., Moser C. (2021). Anti-Biofilm Approach in Infective Endocarditis Exposes New Treatment Strategies for Improved Outcome. Front. Cell Dev. Biol..

[6] Martin I., Waters V., Grasemann H. (2021). Approaches to Targeting Bacterial Biofilms in Cystic Fibrosis Airways. Int. J. Mol. Sci..

[7] Flemming H.-C., Wuertz S. (2019). Bacteria and Archaea on Earth and Their Abundance in Biofilms. Nat. Rev. Microbiol..

[8] Varma A., Warghane A., Dhiman N.K., Paserkar N., Upadhye V., Modi A., Saini R. (2023). The Role of Nanocomposites against Biofilm Infections in Humans. Front. Cell. Infect. Microbiol..

[9] Rather M.A., Gupta K., Mandal M. (2021). Microbial Biofilm: Formation, Architecture, Antibiotic Resistance, and Control Strategies. Braz. J. Microbiol..

[10] Sha M., Xu W., Wu Z., Gu W., Zhu C. (2022). Recent Advances in Single-Atom Materials for Enzyme-Like Catalysis and Biomedical Applications. Chem. J. Chin. Univ.-Chin..

[11] Maduna L., Patnaik A. (2023). A Review of Wound Dressings Treated with Aloe Vera and Its Application on Natural Fabrics. J. Nat. Fibers.

[12] Ortiz Y., Garcia-Heredia A., Merino-Mascorro A., Garcia S., Solis-Soto L., Heredia N. (2021). Natural and Synthetic Antimicrobials Reduce Adherence of Enteroaggregative and Enterohemorrhagic *Escherichia coli* to Epithelial Cells. PLoS ONE.

[13] Kalsy M., Tonk M., Hardt M., Dobrindt U., Zdybicka-Barabas A., Cytrynska M., Vilcinskas A., Mukherjee K. (2020). The Insect Antimicrobial Peptide Cecropin a Disrupts Uropathogenic Escherichia Coli Biofilms. NPJ Biofilms Microbiomes.

[14] Portelinha J., Angeles-Boza A.M. (2021). The Antimicrobial Peptide Gad-1 Clears *Pseudomonas aeruginosa* Biofilms under Cystic Fibrosis Conditions. ChemBioChem.

[15] Liu W., Wu Z., Mao C., Guo G., Zeng Z., Fei Y., Wan S., Peng J., Wu J. (2020). Antimicrobial Peptide Cec4 Eradicates the Bacteria of Clinical Carbapenem-Resistant *Acinetobacter baumannii* biofilm. Front. Microbiol..

[16] Prior B.S., Lange M.D., Salger S.A., Reading B.J., Peatman E., Beck B.H. (2022). The Effect of Piscidin Antimicrobial Peptides on the Formation of Gram-Negative Bacterial Biofilms. J. Fish Dis..

[17] Zhang Y., Cheng P., Wang S., Li X., Peng L., Fang R., Xiong J., Li H., Mei C., Gao J. (2022). *Pseudomonas aeruginosa* Biofilm Dispersion by the Mouse Antimicrobial Peptide Cramp. Vet. Res..

[18] Yan Y., Li Y., Zhang Z., Wang X., Niu Y., Zhang S., Xu W., Ren C. (2021). Advances of Peptides for Antibacterial Applications. Colloid Surf. B—Biointerfaces.

[19] Fu J., Shen T., Wu J., Wang C. (2021). Nanozyme: A New Strategy Combating Bacterial. J. Inorg. Mater..

[20] Yue L., Wang Z., Zheng M., Wang M., Khan I.M., Ding X., Zhang Y. (2022). Water-Soluble Chlorin e6-Hydroxypropyl Chitosan as a High-Efficiency Photoantimicrobial Agent against *Staphylococcus aureus*. Int. J. Biol. Macromol..

[21] Yougbare S., Mutalik C., Krisnawati D.I., Kristanto H., Jazidie A., Nuh M., Cheng T.-M., Kuo T.-R. (2020). Nanomaterials for the Photothermal Killing of Bacteria. Nanomaterials.

[22] Ran T., Ning Z., Liangliang Z., Habumugisha T., Yicun C., Yin L., Yinjuan W., Kui W., Yangdong W., Jianchun J. (2023). Characterization and Antivibrio Activity of Chitosan-Citral Schiff Base Calcium Complex for a Calcium Citrate Sustained Release Antibacterial Agent. Int. J. Biol. Macromol..

[23] Liu Y., Lixuan R., Yanzhen Z., Siqun L., Huifang W., Xianghua G., Baolong N., Wenfeng L. (2023). Preparation and Characterization of PVA/Arginine Chitosan/ZnO NPs Composite Films. Int. J. Biol. Macromol..

[24] Janani B., Okla M.K., Abdel-Maksoud M.A., AbdElgawad H., Thomas A.M., Raju L.L., Al-Qahtani W.H., Khan S.S. (2022). CuO Loaded ZnS Nanoflower Entrapped on PVA-Chitosan Matrix for Boosted Visible Light Photocatalysis for Tetracycline Degradation and Anti-Bacterial Application. J. Environ. Manag..

[25] Zhang J., Sun B., Zhang M., Su Y., Xu W., Sun Y., Jiang H., Zhou N., Shen J., Wu F. (2023). Modulating the Local Coordination Environment of Cobalt Single-Atomic Nanozymes for Enhanced Catalytic Therapy against Bacteria. Acta Biomater..

[26] Shehabeldine A.M., Al-Askar A.A., AbdElgawad H., Hagras F.A., Ramadan A.A., Kamel M.R., Ahmed M.A., Atia K.H., Hashem A.H. (2023). Wound Dressing Scaffold with High Anti-Biofilm Performance Based on Ciprofloxacin-Loaded Chitosan-Hydrolyzed Starch Nanocomposite: In Vitro and in Vivo Study. Appl. Biochem. Biotechnol..

[27] Peng X., Han Q., Zhou X., Chen Y., Huang X., Guo X., Peng R., Wang H., Peng X., Cheng L. (2022). Effect of pH-Sensitive Nanoparticles on Inhibiting Oral Biofilms. Drug Deliv..

[28] Liu D., Xi Y., Yu S., Yang K., Zhang F., Yang Y., Wang T., He S., Zhu Y., Fan Z. (2023). A Polypeptide Coating for Preventing Biofilm on Implants by Inhibiting Antibiotic Resistance Genes. Biomaterials.

[29] Zmejkoski D.Z., Zdravkovic N.M., Trisic D.D., Budimir M.D., Markovic Z.M., Kozyrovska N.O., Markovic B.M.T. (2021). Chronic Wound Dressings-Pathogenic Bacteria Anti-Biofilm Treatment with Bacterial Cellulose-Chitosan Polymer or Bacterial Cellulose-Chitosan Dots Composite Hydrogels. Int. J. Biol. Macromol..

[30] Mohamed A.A., Abu-Elghait M., Ahmed N.E., Salem S.S. (2021). Eco-Friendly Mycogenic Synthesis of ZnO and CuO Nanoparticles for in Vitro Antibacterial, Antibiofilm, and Antifungal Applications. Biol. Trace Elem. Res..

[31] Velgosova O., Mudra E., Vojtko M. (2021). Preparing, Characterization and Anti-Biofilm Activity of Polymer Fibers Doped by Green Synthesized AgNPs. Polymers.

[32] Adnan R., Abdallah A.M., Mezher M., Noun M., Khalil M., Awad R. (2023). Impact of Mg-Doping on the Structural, Optical, and Magnetic Properties of CuO Nanoparticles and Their Antibiofilm Activity. Phys. Scr..

[33] Wang W., Luo Q., Li J., Li L., Li Y., Huo X., Du X., Li Z., Wang N. (2022). Photothermal-Amplified Single Atom Nanozyme for Biofouling Control in Seawater. Adv. Funct. Mater..

[34] Wang X., Hu W., Xia X.-H., Wang C. (2023). Implanting of Single Zinc Sites into 2D Metal-Organic Framework Nanozymes for Boosted Antibiofilm Therapy. Adv. Funct. Mater..

[35] He J., Hong M., Xie W., Chen Z., Chen D., Xie S. (2022). Progress and Prospects of Nanomaterials against Resistant Bacteria. J. Control. Release.

[36] Zhang J., Tang W., Zhang X., Song Z., Tong T. (2023). An Overview of Stimuli-Responsive Intelligent Antibacterial Nanomaterials. Pharmaceutics.

[37] Zhao Z., Ukidve A., Krishnan V., Mitragotri S. (2019). Effect of Physicochemical and Surface Properties on in Vivo Fate of Drug Nanocarriers. Adv. Drug Deliv. Rev..

[38] Xie Y.-Y., Zhang Y.-W., Liu X.-Z., Ma X.-F., Qin X.-T., Jia S.-R., Zhong C. (2021). Aggregation-Induced Emission-Active Amino Acid/Berberine Hydrogels with Enhanced Photodynamic Antibacterial and Anti-Biofilm Activity. Chem. Eng. J..

[39] Shi J., Wang Y., He W., Ye Z., Liu M., Zhao Z., Lam J.W.Y., Zhang P., Kwok R.T.K., Tang B.Z. (2023). Precise Molecular Engineering of Type I Photosensitizer with Aggregation-Induced Emission for Image-Guided Photodynamic Eradication of Biofilm. Molecules.

[40] Wang C., Zhao W., Cao B., Wang Z., Zhou Q., Lu S., Lu L., Zhan M., Hu X. (2020). Biofilm-Responsive Polymeric Nanoparticles with Self-Adaptive Deep Penetration for in Vivo Photothermal Treatment of Implant Infection. Chem. Mat..

[41] Bernal-Mercado A.T., Juarez J., Valdez M.A., Ayala-Zavala J.F., Del-Toro-Sanchez C.L., Encinas-Basurto D. (2022). Hydrophobic Chitosan Nanoparticles Loaded with Carvacrol against *Pseudomonas aeruginosa* Biofilms. Molecules.

[42] Nwabuife J.C., Pant A.M., Govender T. (2021). Liposomal Delivery Systems and Their Applications against *Staphylococcus aureus* and Methicillin-Resistant *Staphylococcus aureus*. Adv. Drug Deliv. Rev..

[43] Karim A.A., Dou Q., Li Z., Loh X.J. (2016). Emerging Supramolecular Therapeutic Carriers Based on Host-Guest Interactions. Chem.—Asian J..

[44] Zhang P., Qian X., Zhang Z., Li C., Xie C., Wu W., Jiang X. (2017). Supramolecular Amphiphilic Polymer-Based Micelles with Seven-Armed Polyoxazoline Coating for Drug Delivery. ACS Appl. Mater. Interfaces.

[45] Gao Q., Huang D., Deng Y., Yu W., Jin Q., Ji J., Fu G. (2021). Chlorin e6 (Ce6)-Loaded Supramolecular Polypeptide Micelles with Enhanced Photodynamic Therapy Effect against *Pseudomonas aeruginosa*. Chem. Eng. J..

[46] Chen M., Qiu B., Zhang Z., Xie S., Liu Y., Xia T., Li X. (2021). Light-Triggerable and Ph/Lipase-Responsive Release of Antibiotics and Β-Lactamase Inhibitors from Host-Guest Self-Assembled Micelles to Combat Biofilms and Resistant Bacteria. Chem. Eng. J..

[47] Shi Y., Cao Y., Cheng J., Yu W., Liu M., Yin J., Huang C., Liang X., Zhou H., Liu H. (2022). Construction of Self-Activated Nanoreactors for Cascade Catalytic Anti-Biofilm Therapy Based on H_2_O_2_ Self-Generation and Switch-on NO Release. Adv. Funct. Mater..

[48] Raj V., Kim Y., Kim Y.-G., Lee J.-H., Lee J. (2022). Chitosan-Gum Arabic Embedded Alizarin Nanocarriers Inhibit Biofilm Formation of Multispecies Microorganisms. Carbohydr. Polym..

[49] Zou L., Hu D., Wang F., Jin Q., Ji J. (2021). The Relief of Hypoxic Microenvironment Using an O_2_ Self-Sufficient Fluorinated Nanoplatform for Enhanced Photodynamic Eradication of Bacterial Biofilms. Nano Res..

[50] Wu S., Xu C., Zhu Y., Zheng L., Zhang L., Hu Y., Yu B., Wang Y., Xu F.-J. (2021). Biofilm-Sensitive Photodynamic Nanoparticles for Enhanced Penetration and Antibacterial Efficiency. Adv. Funct. Mater..

[51] Gao Y., Wang J., Chai M., Li X., Deng Y., Jin Q., Ji J. (2020). Size and Charge Adaptive Clustered Nanoparticles Targeting the Biofilm Microenvironment for Chronic Lung Infection Management. ACS Nano.

[52] Ding M., Zhao W., Zhang X., Song L., Luan S. (2022). Charge-Switchable MOF Nanocomplex for Enhanced Biofilm Penetration and Eradication. J. Hazard. Mater..

[53] Yuan Z., Lin C., He Y., Tao B., Chen M., Zhang J., Liu P., Cai K. (2020). Near-Infrared Light-Triggered Nitric-Oxide-Enhanced Photodynamic Therapy and Low-Temperature Photothermal Therapy for Biofilm Elimination. ACS Nano.

[54] Fu H., Xue K., Zhang Y., Xiao M., Wu K., Shi L., Zhu C. (2023). Thermoresponsive Hydrogel-Enabled Thermostatic Photothermal Therapy for Enhanced Healing of Bacteria-Infected Wounds. Adv. Sci..

[55] Cai X., Tian J., Zhu J., Chen J., Li L., Yang C., Chen J., Chen D. (2021). Photodynamic and Photothermal co-Driven CO-Enhanced Multi-Mode Synergistic Antibacterial Nanoplatform to Effectively Fight against Biofilm Infections. Chem. Eng. J..

[56] Vidakis N., Petousis M., Michailidis N., Papadakis V., Korlos A., Mountakis N., Argyros A. (2022). Multi-Functional 3D-Printed Vat Photopolymerization Biomedical-Grade Resin Reinforced with Binary Nano Inclusions: The Effect of Cellulose Nanofibers and Antimicrobial Nanoparticle Agents. Polymers.

[57] Zhou C., Zhou Y., Zheng Y., Yu Y., Yang K., Chen Z., Chen X., Wen K., Chen Y., Bai S. (2023). Amphiphilic Nano-Swords for Direct Penetration and Eradication of Pathogenic Bacterial Biofilms. ACS Appl. Mater. Interfaces.

[58] Wan P., Guo W., Wang Y., Deng M., Xiao C., Chen X. (2022). Photosensitizer-Polypeptide Conjugate for Effective Elimination of *Candida albicans* Biofilm. Adv. Healthc. Mater..

[59] Mustafa Y.F. (2023). Modern Developments in the Application and Function of Metal/Metal Oxide Nanocomposite-Based Antibacterial Agents. BioNanoScience.

[60] Maric T., Lovind A., Zhang Z., Geng J., Boisen A. (2023). Near-Infrared Light-Driven Mesoporous SiO_2_/Au Nanomotors for Eradication of *Pseudomonas aeruginosa* Biofilm. Adv. Healthc. Mater..

[61] Zheng X., Sun J., Li W., Dong B., Song Y., Xu W., Zhou Y., Wang L. (2020). Engineering Nanotubular Titania with Gold Nanoparticles for Antibiofilm Enhancement and Soft Tissue Healing Promotion. J. Electroanal. Chem..

[62] Cao M., Chang Z., Tan J., Wang X., Zhang P., Lin S., Liu J., Li A. (2022). Superoxide Radical-Mediated Self-Synthesized Au/MoO_(3-X)_ Hybrids with Enhanced Peroxidase-Like Activity and Photothermal Effect for Anti-Mrsa Therapy. ACS Appl. Mater. Interfaces.

[63] Chang M., Wang M., Chen Y., Shu M., Zhao Y., Ding B., Hou Z., Lin J. (2019). Self-Assembled CeVO_4_/Ag Nanohybrid as Photoconversion Agents with Enhanced Solar-Driven Photocatalysis and Nir-Responsive Photothermal/Photodynamic Synergistic Therapy Performance. Nanoscale.

[64] D’Agostino A., Taglietti A., Desando R., Bini M., Patrini M., Dacarro G., Cucca L., Pallavicini P., Grisoli P. (2017). Bulk Surfaces Coated with Triangular Silver Nanoplates: Antibacterial Action Based on Silver Release and Photo-Thermal Effect. Nanomaterials.

[65] Gonzalez-Fernandez S., Lozano-Iturbe V., Garcia B., Andres L.J., Menendez M.F., Rodriguez D., Vazquez F., Martin C., Quiros L.M. (2020). Antibacterial Effect of Silver Nanorings. BMC Microbiol..

[66] Kim J.-H., Ma J., Jo S., Lee S., Kim C.S. (2020). Enhancement of Antibacterial Performance of Silver Nanowire Transparent Film by Post-Heat Treatment. Nanomaterials.

[67] Bi X., Bai Q., Liang M., Yang D., Li S., Wang L., Liu J., Yu W.W., Sui N., Zhu Z. (2022). Silver Peroxide Nanoparticles for Combined Antibacterial Sonodynamic and Photothermal Therapy. Small.

[68] Obeng E., Feng J., Wang D., Zheng D., Xiang B., Shen J. (2022). Multifunctional Phototheranostic Agent ZnO@Ag for Anti-Infection through Photothermal/Photodynamic Therapy. Front. Chem..

[69] Elyamny S., Eltarahony M., Abu-Serie M., Nabil M.M., Kashyout A.E.-H.B. (2021). One-Pot Fabrication of Ag@Ag_2_O Core-Shell Nanostructures for Biosafe Antimicrobial and Antibiofilm Applications. Sci. Rep..

[70] Ye L., Cao Z., Liu X., Cui Z., Li Z., Liang Y., Zhu S., Wu S. (2022). Noble Metal-Based Nanomaterials as Antibacterial Agents. J. Alloys Compd..

[71] Sonbol H., Ameen F., AlYahya S., Almansob A., Alwakeel S. (2021). *Padina boryana* Mediated Green Synthesis of Crystalline Palladium Nanoparticles as Potential Nanodrug against Multidrug Resistant Bacteria and Cancer Cells. Sci. Rep..

[72] Zhou Y., Zhou Z., Wu X., Wang Z., Qi W., Yang J., Qing L., Tang J., Deng L. (2023). Down-Regulation of Hsp by Pd-Cu Nanozymes for Nir Light Triggered Mild-Temperature Photothermal Therapy against Wound Bacterial Infection: In Vitro and in Vivo Assessments. Int. J. Nanomed..

[73] Khan F., Lee J.-W., Pham D.N.T., Khan M.M., Park S.-K., Shin I.-S., Kim Y.-M. (2020). Antibiofilm Action of ZnO, SnO_2_ and CeO_2_ Nanoparticles towards Gram-Positive Biofilm Forming Pathogenic Bacteria. Recent Pat. Nanotechnol..

[74] Togawa G., Takahashi M., Tada H., Takada Y. (2022). Development of Ternary Ti-Ag-Cu Alloys with Excellent Mechanical Properties and Antibiofilm Activity. Materials.

[75] Chen Q., Qi M., Shi F., Liu C., Shi Y., Sun Y., Bai X., Wang L., Sun X., Dong B. (2023). Novel Twin-Crystal Nanosheets with MnO_2_ Modification to Combat Bacterial Biofilm against Periodontal Infections via Multipattern Strategies. Adv. Healthc. Mater..

[76] Aziz S.N., Al-Kadmy I.M.S., Rheima A.M., Al-Sallami K.J., Abd Ellah N.H., El-Saber Batiha G., El-Bouseary M.M., Algammal A.M., Hetta H.F. (2023). Binary CuO\CoO Nanoparticles Inhibit Biofilm Formation and Reduce the Expression of Papc and Fimh Genes in Multidrug-Resistant Klebsiella Oxytoca. Mol. Biol. Rep..

[77] Leung Y.H., Xu X., Ma A.P.Y., Liu F., Ng A.M.C., Shen Z., Gethings L.A., Guo M.Y., Djurisic A.B., Lee P.K.H. (2016). Toxicity of ZnO and TiO_2_ to Escherichia Coli Cells. Sci. Rep..

[78] Yuan G., Zhang S., Yang Z., Wu S., Chen H., Tian X., Cheng S., Pan Y., Zhou R. (2022). Precisely Modulated 2D PdCu Alloy Nanodendrites as Highly Active Peroxidase Mimics for the Elimination of Biofilms. Biomater. Sci..

[79] Li Y., Yang L., Liao Y., Zhao R., Ji L., Su R., Xu D., Wang F. (2023). Photothermal Heating-Assisted Superior Antibacterial and Antibiofilm Activity of High-Entropy-Alloy Nanoparticles. Adv. Funct. Mater..

[80] Mamani J.B., Borges J.P., Rossi A.M., Gamarra L.F. (2023). Magnetic Nanoparticles for Therapy and Diagnosis in Nanomedicine. Pharmaceutics.

[81] Liu W., Pei W., Moradi M., Zhao D., Li Z., Zhang M., Xu D., Wang F. (2022). Polyethyleneimine Functionalized Mesoporous Magnetic Nanoparticles with Enhanced Antibacterial and Antibiofilm Activity in an Alternating Magnetic Field. ACS Appl. Mater. Interfaces.

[82] Ji Y., Han Z., Ding H., Xu X., Wang D., Zhu Y., An F., Tang S., Zhang H., Deng J. (2021). Enhanced Eradication of Bacterial/Fungi Biofilms by Glucose Oxidase-Modified Magnetic Nanoparticles as a Potential Treatment for Persistent Endodontic Infections. ACS Appl. Mater. Interfaces.

[83] Mutalik C., Saukani M., Khafid M., Krisnawati D.I., Widodo R., Darmayanti R., Puspitasari B., Cheng T.-M., Kuo T.-R. (2023). Gold-Based Nanostructures for Antibacterial Application. Int. J. Mol. Sci..

[84] Yin M., Qiao Z., Yan D., Yang M., Yang L., Wan X., Chen H., Luo J., Xiao H. (2021). Ciprofloxacin Conjugated Gold Nanorods with pH Induced Surface Charge Transformable Activities to Combat Drug Resistant Bacteria and Their Biofilms. Mater. Sci. Eng. C-Mater. Biol. Appl..

[85] Aguilera-Correa J.J., Garcia-Alvarez R., Mediero A., Esteban J., Vallet-Regi M. (2022). Effect of Gold Nanostars plus Amikacin against Carbapenem-Resistant *Klebsiella pneumoniae* Biofilms. Biology.

[86] He X., Dai L., Ye L., Sun X., Enoch O., Hu R., Zan X., Lin F., Shen J. (2022). A Vehicle-Free Antimicrobial Polymer Hybrid Gold Nanoparticle as Synergistically Therapeutic Platforms for *Staphylococcus aureus* Infected Wound Healing. Adv. Sci..

[87] Tang Y.Z., Wang T.J., Feng J.H., Rong F., Wang K., Li P., Huang W. (2021). Photoactivatable Nitric Oxide-Releasing Gold Nanocages for Enhanced Hyperthermia Treatment of Biofilm-Associated Infections. ACS Appl. Mater. Interfaces.

[88] Zhang M., Zhang H., Feng J., Zhou Y., Wang B. (2020). Synergistic Chemotherapy, Physiotherapy and Photothermal Therapy against Bacterial and Biofilms Infections through Construction of Chiral Glutamic Acid Functionalized Gold Nanobipyramids. Chem. Eng. J..

[89] Liu C., Wang J., Wan J., Yu C. (2021). MOF-on-MOF Hybrids: Synthesis and Applications. Coord. Chem. Rev..

[90] He Y., Wang X., Zhang C., Sun J., Xu J., Li D. (2023). Near-Infrared Light-Mediated Cyclodextrin Metal-Organic Frameworks for Synergistic Antibacterial and Anti-Biofilm Therapies. Small.

[91] Fan X., Wu X., Yang F., Wang L., Ludwig K., Ma L., Trampuz A., Cheng C., Haag R. (2022). A Nanohook-Equipped Bionanocatalyst for Localized Near-Infrared-Enhanced Catalytic Bacterial Disinfection. Angew. Chem. Int. Ed..

[92] Concepcion O., de Melo O. (2023). The Versatile Family of Molybdenum Oxides: Synthesis, Properties, and Recent Applications. J. Phys.—Condens. Matter.

[93] Obaid N.M., Al-Nafiey A., Al-Dahash G. (2023). Graphene/Molybdenum Disulfide Nanocomposites: Characterization and Optoelectronic Application. J. Nanophoton..

[94] Zhang Y., Wang Y., Guo C., Wang Y. (2022). Molybdenum Carbide-Based Photocatalysts: Synthesis, Functionalization, and Applications. Langmuir.

[95] Li H., Gong M., Xiao J., Hai L., Luo Y., He L., Wang Z., Deng L., He D. (2022). Photothermally Activated Multifunctional MoS_2_ Bactericidal Nanoplatform for Combined Chemo/Photothermal/Photodynamic Triple-Mode Therapy of Bacterial and Biofilm Infections. Chem. Eng. J..

[96] Li H., Yang K., Hai L., Wang Z., Luo Y., He L., Yi W., Li J., Xu C., Deng L. (2023). Photothermal-Triggered Release of Alkyl Radicals and Cascade Generation of Hydroxyl Radicals via a Versatile Hybrid Nanocatalyst for Hypoxia-Irrelevant Synergistic Antibiofilm Therapy. Chem. Eng. J..

[97] Tasia W., Lei C., Cao Y., Ye Q., He Y., Xu C. (2020). Enhanced Eradication of Bacterial Biofilms with DNase I-Loaded Silver-Doped Mesoporous Silica Nanoparticles. Nanoscale.

[98] Mohammadpour R., Dobrovolskaia M.A., Cheney D.L., Greish K.F., Ghandehari H. (2019). Subchronic and Chronic Toxicity Evaluation of Inorganic Nanoparticles for Delivery Applications. Adv. Drug Deliv. Rev..

[99] Xia L., Park J.H., Biggs K., Lee C.G., Liao L., Shannahan J.H. (2023). Compositional Variations in Metal Nanoparticle Components of Welding Fumes Impact Lung Epithelial Cell Toxicity. J. Toxicol. Environ. Health Part A.

[100] Dianova L., Tirpak F., Halo M., Slanina T., Massanyi M., Stawarz R., Formicki G., Madeddu R., Massanyi P. (2022). Effects of Selected Metal Nanoparticles (Ag, ZnO, TiO_2_) on the Structure and Function of Reproductive Organs. Toxics.

